# Bovine Genome Database: new curated collection of selective sweeps in bovine populations across the world

**DOI:** 10.1093/nar/gkaf1214

**Published:** 2025-11-20

**Authors:** Sumaya Kambal, Amy T Walsh, Sathesh K Sivasankaran, Nigatu A Adossa, Joseph H Skarlupka, Olivier Hanotte, Garret Suen, Christine G Elsik

**Affiliations:** Division of Animal Sciences, University of Missouri, Columbia, MO 65211, United States; Livestock Genetics, International Livestock Research Institute, Addis Ababa 5689, Ethiopia; Division of Animal Sciences, University of Missouri, Columbia, MO 65211, United States; Division of Animal Sciences, University of Missouri, Columbia, MO 65211, United States; Department of Neurology, Washington University in St. Louis, St. Louis, MO 63108, United States; Neurogenomics and Informatics Center, Washington University School of Medicine, St. Louis, MO 63108, United States; Livestock Genetics, International Livestock Research Institute, Addis Ababa 5689, Ethiopia; Microbiology Doctoral Training Program, University of Wisconsin–Madison, Madison, WI 53706, United States; Department of Bacteriology, University of Wisconsin–Madison, Madison, WI 53706, United States; Livestock Genetics, International Livestock Research Institute, Addis Ababa 5689, Ethiopia; Centre for Tropical Livestock Genetics and Health, International Livestock Research Institute, Addis Ababa 5689, Ethiopia; Cells, Organisms and Molecular Genetics, School of Life Sciences, University of Nottingham, Nottingham NG7 2RD, United Kingdom; Department of Bacteriology, University of Wisconsin–Madison, Madison, WI 53706, United States; Division of Animal Sciences, University of Missouri, Columbia, MO 65211, United States; Division of Plant Sciences & Technology, University of Missouri, Columbia, MO 65211, United States; MU Institute for Data Science & Informatics, University of Missouri, Columbia, MO 65211, United States

## Abstract

Mapping genome-wide selective sweeps is of high relevance in cattle population genomics, having successfully identified thousands of genomic regions and candidate genes, with potential to reveal links to agriculturally important traits such as those related to production and adaptation to extreme environments. However, pinpointing the underlying causal variants remains a key priority in understanding molecular mechanisms controlling these traits. The lack of an integrative resource for selective sweeps has impeded meta-analysis and candidate variant prioritization. In the current update of the Bovine Genome Database (BGD; https://bovinegenome.elsiklab.missouri.edu), we address this gap by incorporating a curated dataset consolidating 92 519 selective sweeps identified through 340 genome-wide analyses across 213 cattle populations worldwide. Incorporating this new dataset into BovineMine enables meta-analysis across studies and populations to identify consensus signals, and the exploration of selective sweeps in the context of genes, gene functions, genomic variations, and quantitative trait loci. Furthermore, the BGD JBrowse genome browser enables visualization of sweep regions alongside other genomic features and functional annotations such as histone marks, open chromatin regions, and chromatin states. This BGD update facilitates the prioritization of candidate causal variants and helps identify unanswered questions in disentangling the molecular basis of adaptive and economically important traits in cattle.

## Graphical Abstract

**Graphical Abstract:**
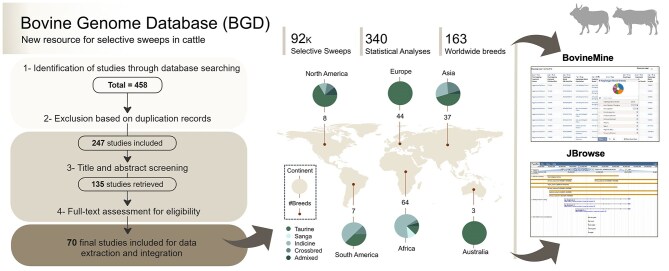

## Introduction

The identification of genetic markers and causal variants underlying agriculturally important traits is a primary goal in bovine genomics and a critical priority towards developing genetic improvement programs for cattle breeds worldwide. Notably, advances in sequencing and omics technologies, coupled with their reduced cost, have significantly accelerated the generation of high-quality genomic resources. This increasing amount of data, while supportive for genomic-based research, has also created an urgent need for data-mining resources necessary to enable effective usage of these datasets and make integrative analysis of new findings with existing biological information, such as gene annotation and function, more powerful. The continuous improvements to the Bovine Genome Database (BGD; https://bovinegenome.elsiklab.missouri.edu) since its establishment [1–3] have provided the research community with indispensable tools for data mining, genome navigation, and annotation with accessible catalogues of various biological features. These include, but are not limited to, genes (coding and non-coding), gene expression, Gene Ontology (GO) [4, 5], genomic variation, repetitive elements, and quantitative trait loci (QTL). This has empowered researchers to elucidate core driver genes identified as potential candidates involved in biological pathways related to several traits of interest in cattle breeds [6–12]. Moreover, the integrative visualization for multiple lines of evidence through BGD tools has enabled scientists to test and formulate new hypotheses regarding gene regulation and function.

Toward enhanced understanding of key traits, researchers are increasingly mapping the genomic footprints of selection, also known as selective sweeps, in different breeds and populations from across the world, providing insights into how genomes have responded to natural and human-driven selection pressures (see reviews [13–16]). By investigating patterns left by these selection episodes, such as reduced genetic diversity and extended haplotype homozygosity, studies have identified candidate genes and genomic variants that potentially exhibit selective advantages linked to economically important traits such as milk yield and quality, disease tolerance, and adaptation to extreme environments, e.g. [17–20]. However, while thousands of genomic regions under selection have been identified, often overlapping non-coding regulatory elements, the causal variants and their molecular mechanisms of action remain largely unknown. This gap highlights the need for prioritization strategies to identify high-confidence candidate genes and variants. To address this, we have created a new dataset of compiled selective sweeps reported in domesticated cattle breeds. We have incorporated this resource into the BovineMine data mining tool to empower meta-analyses of selection sweeps across studies and populations, facilitating the identification of consensus signals and their integration with other functional annotation data categories. Exploring this consolidated data will lead to hypotheses aimed at validating candidate variants in different populations, ultimately filling in gaps in knowledge required to design efficient genomic selection breeding programs.

## New selective sweep resource

**Figure 1.**
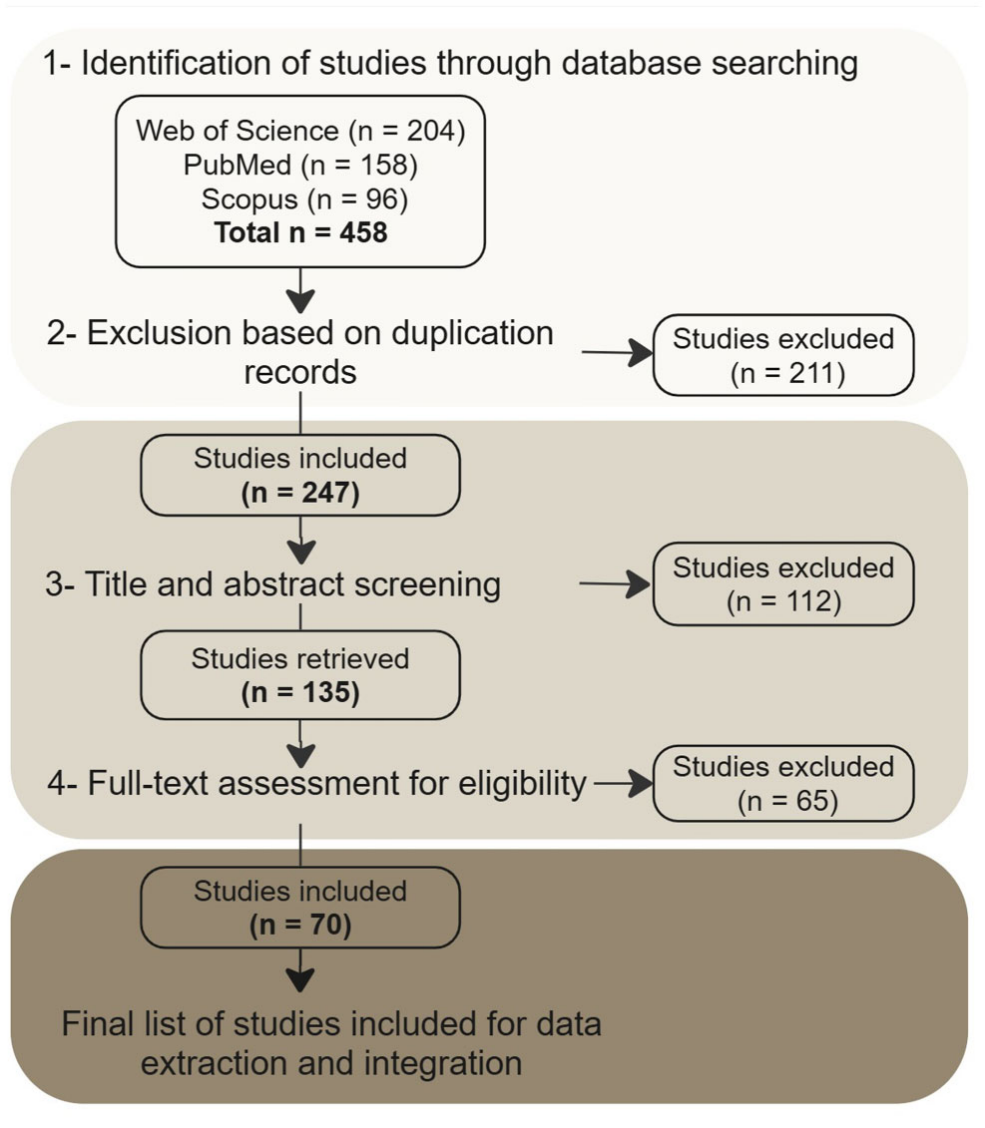
A flowchart showing the process for selecting published studies to include in the selective sweep resource.

To construct a comprehensive resource, we conducted a systematic review of available literature to identify relevant studies reporting genome-wide selective sweeps in cattle. We queried three major electronic databases, PubMed, Web of Science, and Scopus (as of 13 June 2025), using a combination of keywords including [(“signatures of selection” OR “selective signature” OR “selection sweep” OR “sweeps” OR “positive selection” OR “balanced selection” OR “selection”) AND (“cattle” OR “domesticated cattle” OR “Bos taurus” OR “Bos indicus” OR “taurine” OR “indicine”)]. The initial search yielded a total of 458 studies, which were then screened based on title/abstract and full text (Fig. 1). We included studies based on different criteria, mainly if they: (i) analyzed genome-wide data from domesticated cattle breeds (*Bos taurus, Bos indicus*, crossbred, sanga, and other admixed); (ii) identified selective sweeps using defined statistical methods (Supplementary Table S1 in Supplementary File 1) [21–48]; and (iii) reported genomic coordinates of sweep regions, including both start and end (see Supplementary Table S2 in Supplementary File 1 for detailed criteria). The final set comprises 70 articles [17–20, 36, 49–113]. Information related to the selective sweeps was extracted manually from the articles, including breeds, statistical method, test value, and genome assembly version. Some of the articles reported analyses performed per breed, and others combined breeds into groups, often inhabiting similar environments. To facilitate data exploration at BGD, we created descriptive population names based on geographic origin combined with either breed (for single-breed analyses), breed class, or an adaptive quality for a group of breeds described in the article.

When available, genome coordinates were collected from the supplementary files. In a few cases, genome coordinates had to be manually curated from tables provided as images. For studies performed using the UMD3.1 bovine reference assembly, we used the UCSC LiftOver tool [114] and a chain file downloaded from the UCSC Genome Browser [115] to transfer coordinates to the ARS-UCD1.2 genome assembly. The ARS-UCD1.2 assembly is identical to the current ARS-UCD2.0 assembly, except the former is lacking a Y chromosome and the latter is lacking 254 unassigned scaffolds that had been previously determined to be contaminants. The assembly differences between ARS-UCD1.2 and ARS-UCD2.0 did not affect the selective sweep regions, because the regions are all located on assembled chromosomes.

Details on the selected studies are provided in Supplementary File 2. The final dataset encompasses 92 519 selective sweeps detected through 340 independent genome-wide analyses across 213 population groups representing 163 breeds classified as taurine (*Bos taurus, n* = 83), indicine (*Bos indicus, n* = 56), crossbred (*n* = 9), sanga (*n* = 8), and other admixed cattle (*n* = 7). Across all studies, we noticed that many analyses were combined with publicly available data from previous work to increase statistical power. A particular challenge for studies employing complex pairwise population comparisons was a lack of metadata regarding the target population of reported sweeps. Therefore, sweeps for which we could not verify the population were excluded.

To prepare the data for incorporation into BovineMine and JBrowse, each selective sweep region from each analysis was assigned a unique identifier with the prefix BOVSS followed by six digits. The unique identifiers allowed each region to be a data object with attributes for population, breed, breed class, breed origin, number of target breeds, publication, statistical test, and statistical value (when available). Although all the curated sweeps were identified as being significant based on the published criteria and thresholds, we assigned “NO VALUE” if a specific value for each sweep was not provided in the published article. Consequently, the dataset enables statistical metric-based filtration where statistical values are available, but missing values represent a gap that may challenge future meta-analysis.

**Figure 2.**
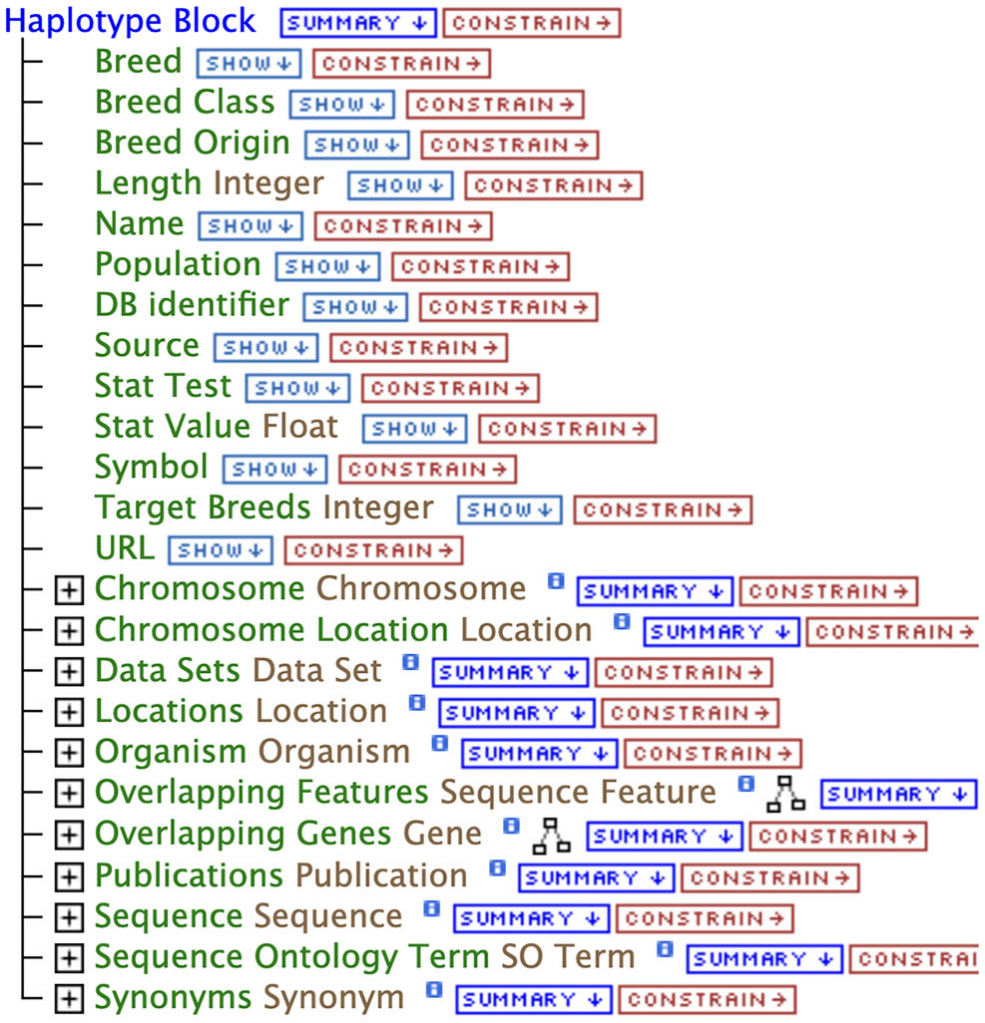
A BovineMine QueryBuilder view showing the data model, rooted at “Haplotype Block,” which is the Sequence Ontology term for selective sweep regions used in BovineMine. Connected to the root term are the attributes (from Breed to Publication URL). Below those, with plus signs, are data collections that are connected to the selective sweep data. The collection of overlapping genes was pre-computed to enable fast queries. The “Overlapping Features” collection contains all data types that have genome coordinates, including QTL and variants, and these have been computed by InterMine.

Figure 2 shows the data model for the selective sweep data collection, as depicted by the BovineMine QueryBuilder. The data model is represented as a tree rooted at the class “Haplotype Block.” Because InterMine’s core biological model is based on the Sequence Ontology (SO) [116, 117], we are constrained to use existing SO terms, available at http://www.sequenceontology.org/, for all genomic features. We chose the SO term “haplotype_block” to represent the selective sweep regions. As a result, the QueryBuilder, query prompts, and column headings in BovineMine are labeled “Haplotype Block” rather than “Selective Sweep.” Explanations of the use of the term “Haplotype Block” are provided in the query menus as well as the Regions Search page.

We have created 16 new template queries for the selective sweep data collection, and these are available under the Variation tab in the Templates bar in the middle of the BovineMine home page. The template queries, which include queries that integrate the selective sweeps with genes and QTL, provide an easy way to explore the data. Selective sweep regions can also be retrieved using the Regions Search by entering genome coordinates and selecting “Haplotype Block” as the feature type. The selective sweep data is also available for viewing in JBrowse (Supplementary Figs S1 and S2 in Supplementary File 1) and downloading in gff3 format.

## Other BGD updates

The primary tools available at BGD are the BovineMine genomic data mining warehouse [2, 118], developed using the InterMine platform [117], and the JBrowse [119] genome browser integrated with Apollo [120] annotation and BLAST sequence search [121, 122] tools. These tools have all been updated with the ARS-UCD2.0 genome assembly, which includes the bovine Y chromosome [123, 124].

We have recomputed RNA-seq-based gene expression levels for the RNA-seq data that were previously available at BGD [125]. We have also added 428 new RNA-seq experiments from six BioProjects [126–131] that were selected because they were generated by the Functional Annotation of Animal Genomes Consortium (FAANG). Our methods for computing JBrowse tracks and BovineMine gene expression levels using a Hisat2/StringTie2 [132, 133] pipeline as previously reported [3]. In addition, 23 previously available long-read transcriptome datasets (Iso-Seq) [125] were remapped to ARS-UCD2.0 using minimap2 version 2.2.4 [134] and assembled with StringTie version 2.2.3 to create JBrowse tracks. An additional 103 tissue-specific tracks show chromatin states and peaks from ATAC-seq and ChIP-seq experiments generated by a FAANG project [130].

We curated metadata for the tissue-specific transcriptome and functional annotation data from the NCBI Sequence Read Archive and BioSamples database [135] and the EMBL-EBI BioSamples database [136], expanding the expression metadata compared to previous BovineMine releases to include additional ontology terms and sample attributes. New ontologies describing the samples include the Cell Ontology [137] and the Uberon Anatomy Ontology [138], depending on the sample type. We manually curated Uberon terms for samples that did not already have assigned terms in EMBL-EBI BioSamples. We also manually curated BRENDA Tissue Ontology [139] terms, previously used to describe samples at BGD, for all new samples. To facilitate selection of the tissue-specific tracks in JBrowse, we assigned the tracks to broad categories based on organ systems determined using the Uberon Anatomy Ontology.

We have also updated datasets from external bioinformatic resources. BovineMine includes genes from Ensembl [140] and RefSeq [141], proteins from UniProt [142], protein domains from InterPro [143], pathways from KEGG [144] and Reactome [145], orthologs from OrthoDB [146] and Ensembl Compara [140], variants and variant effects from Ensembl [140], QTL from AnimalQTLdb [147], SNP array alias identifiers [148], publications from NCBI PubMed [135], and GO annotations from Ensembl, RefSeq, and UniProt [140–142]. Version numbers and release dates for these datasets are available on the BovineMine data source page (https://bovinemine.rnet.missouri.edu/bovinemine/dataCategories.do).

## BovineMine application programming interface

The InterMine platform provides a web service API that enables users to automate workflows and access data without using the web app. Web services that are built into InterMine support most of the operations that are available in the web app, with client library support in Python, Perl, Java, JavaScript, Ruby, and R [149, 150], including query templates, custom queries created with the QueryBuilder, and List Upload. In addition, we have developed Python support for the Regions Search and have made BovineMine-specific examples available on GitHub (https://github.com/elsiklab/intermine-api-python-examples/tree/main/bovinemine).

An API key is required to connect to BovineMine and can be acquired on the “Account Details” page under the “MyMine” tab after logging in to BovineMine. This API token is used in place of user credentials in API scripts. You can find information about the Perl, Python, Java, and Ruby API libraries under the API tab in the BovineMine navigation bar. To get started with the API, you can retrieve automatically generated code to run a query by clicking “Perl,” “Python,” “Ruby,” or “Java” in the bar near the bottom of any template query menu or below the “Fields selected for output” section in the QueryBuilder interface.

## Use cases

The integration of the new selective sweep resource into BovineMine enables several alternatives for querying the data. Template queries have been created to search for selective sweeps and associated genomic features such as genes and QTL, and queries can be constrained based on population, breed, QTL trait, QTL identifier, and selective sweep identifier. A simple use case is to query for genes found within selective sweep regions for a selected population. This search is accomplished using the template query “Population → Selected Sweeps and Genes.” The output includes both RefSeq and Ensembl genes, which are saved as lists for use in other queries and for enrichment analyses to identify overrepresented functions. In this Example 1 (Supplementary Figs S3–S11 in Supplementary File 1), we select the population “Highland Bolivian Creole” (Supplementary Figs S3 and S4 in Supplementary File 1) and retrieve 755 RefSeq and 745 Ensembl genes (Supplementary Fig. S6 in Supplementary File 1). We find both gene sets to be enriched for terms related to immune response, response to stimuli, and olfactory reception (Supplementary Figs S10 and S11 in Supplementary File 1 and Supplementary File 3).

Populations can also be investigated to identify QTL located within selective sweeps. In Example 2 (Supplementary Figs S12–S20 in Supplementary File 1), the “Population → Selective Sweeps and QTL” template query is used to identify selective sweeps in a population of the dairy breed, Finnish Ayrshire, that harbor any QTL stored in BovineMine. In the output table you can use the histogram icon above the “Overlapping Features Name” column to view QTL trait names and numbers found (Supplementary Figs S13 and S14 in Supplementary File 1). In this example, we filter for the 1008 rows with selective sweep regions containing “Milk Fat Yield” QTL, and then use the histogram icon above the “Haplotype Block DB Identifiers” column to find that there are 13 selective sweeps overlapping milk fat yield QTL and the majority of the QTL (828) reside in a single selective sweep region (Supplementary Fig. S15 in Supplementary File 1). We then save those 13 selective sweeps as a list and use the saved list in the template query “Selective Sweep (Haplotype Block) ID → Genes” to retrieve genes located within those selective sweep regions (Supplementary Figs S16 andS17 in Supplementary File 1). The genes are enriched for molecular function terms related to small molecule binding and fatty acid binding and for pathways such as linoleic acid metabolism and triglyceride metabolism (Supplementary Figs S19 and S20 in Supplementary File 1 and Supplementary File 3).

Another way to explore the data is to look for populations containing selective sweeps harboring QTL for specific traits. In Example 3 (Supplementary Fig. S21 in Supplementary File 1) we use the template query “QTL Trait → Selective Sweeps and Population Info” to identify populations and breeds with “Aggressive behavior” QTL. We find nine diverse populations with one or more aggressive behavior QTL within selective sweeps.

An alternative use case for the selective sweep resource in BovineMine is to perform meta-analysis with your own research data or data from the literature. In Example 4 (Supplementary Figs S22–S27 in Supplementary File 1) we use genomic coordinates of published body conformation trait QTL in Canadian Holstein Cattle [151]. The coordinates are uploaded using the Regions Search menu to search for overlapping selective sweep regions (Supplementary Fig. S22 in Supplementary File 1). The selective sweep regions found are saved as a list (Supplementary Fig. S23 in Supplementary File 1). On the List Analysis page, we filter the table of selective sweep regions for the 12 populations containing Holstein and save them as another list (Supplementary Figs S24–S26 in Supplementary Table 1). The list is then used in the “Selective Sweep (Haplotype Block) ID → Genes” template query to retrieve 516 genes within those regions (Supplementary Fig. S27 in Supplementary File 1).

## Accessing BGD data and tools

The tools described here are freely available at https://bovinegenome.elsiklab.missouri.edu without registering for an account. However, creating a BovineMine account by clicking “Log in” on the BovineMine home page and working while logged in enables query histories to be saved automatically and allows you to save queries, query templates, and lists for later sessions or for sharing with colleagues.

## Citing BGD

Cite this article for the use of BGD tools such as BovineMine, JBrowse, Apollo, BLAST, and code available on GitHub.

## Conclusions

With the new curated selective sweep resource, updates, and improved tools in BGD, we provide a framework for the research community to synthesize knowledge on signatures of selection in cattle. By incorporating a collection of ~92K genomic regions reported to be under selection in published studies, we directly address the challenges of incomparable findings that have long impeded progress on elucidating the genetic architecture of complex agricultural traits. The resource supports researchers and investigators to move beyond limited single-study observations by enabling powerful meta-analyses that intersect selective sweeps, genes, QTLs, and regulatory annotation. This integrated approach will accelerate the prioritization of candidate variants across populations, facilitating the formulation of biological hypotheses for validation studies. This represents an important step towards designing efficient genomic-based breeding programs for cattle sustainability.

We have described several scenarios for traits believed to have evolved through natural and/or human-driven selection as examples to help users to get started and adjust their query according to their objectives. We encourage the research community to leverage this integrated resource to validate candidate genes and genomic variations. By providing this resource and tools, we aim to fill gaps in knowledge required to drive actionable insights for cattle breeding.

## Supplementary Material

gkaf1214_Supplemental_Files

## Data Availability

The curated selective sweep data are available at the Bovine Genome Database (https://bovinegenome.elsiklab.missouri.edu/downloads/ARS-UCD2.0) and Figshare (https://doi.org/10.6084/m9.figshare.30113080.v1). All external data sources are provided in the BovineMine Data Source page (https://bovinemine.rnet.missouri.edu/bovinemine/dataCategories.do).
