# Supplementary material for: Bovine Genome Database: new curated collection of selective sweeps in bovine populations across the world
Source: Nucleic Acids Res. 2025 Nov 20;54(D1):D949–57. doi: 10.1093/nar/gkaf1214 (PMC12807739; doi:10.1093/nar/gkaf1214)
Supplement: gkaf1214_Supplemental_Files [file gkaf1214_supplemental_files.zip › Supplementary_File_1.pdf]

Supplementary File 1 for

Bovine Genome Database: New Curated Collection of Selective Sweeps in Bovine Populations Across the World

Sumaya Kambal[1,2], Amy T. Walsh[1], Sathesh K. Sivasankaran[1,3,4], Nigatu A. Adossa[2], Joseph H. Skarlupka[5,6], Olivier Hanotte[2,7,8], Garret Suen[6], Christine G. Elsik[1,9,10]

1 Division of Animal Sciences, University of Missouri, Columbia MO 65211, USA
2 Livestock Genetics, International Livestock Research Institute, Addis Ababa, Ethiopia
3 Department of Neurology, Washington University in St. Louis, St. Louis, MO 63108, USA
4 Neurogenomics and Informatics Center, Washington University School of Medicine, St. Louis, MO 63108, USA
5 Microbiology Doctoral Training Program, University of Wisconsin–Madison 53706, Madison WI, USA
6 Department of Bacteriology, University of Wisconsin–Madison, Madison WI 53706, USA
7 Centre for Tropical Livestock Genetics and Health, International Livestock Research Institute, Addis Ababa, Ethiopia
8 School of Life Sciences, University of Nottingham, Nottingham NG7 2UH, UK
9 Division of Plant Sciences & Technology, University of Missouri, Columbia MO 65211, USA
10 MU Institute for Data Science & Informatics, University of Missouri, Columbia, MO 65211, USA

Table S1. Abbreviation and expanded name of statistical tests used in studies included in the selective sweep dataset

Statistical test	Expanded name	Reference*
Alpha	Locus-population specific FST	(1)
CLR	Composite likelihood ratios	(2)
CSS	Composite selection signal	(3)
DCMS	De-correlated composite of multiple selection signals	(4)
delta_AF	Change in allele frequency	(5)
DFL	Fu and Li's D test	(6)
Di	Divergence	(7)
EHH	Extended haplotype homozygosity	(8)
Extended_low_diversity_haplotype	Extended region of reduced diversity	(9)
FST	Fixation index	(10)
hapFLK	Haplotype-based frequency incorporates FLK statistics	(11)
HER	Heterozygosity-enriched region	(12)
HMM	Hidden Markov model	(13)
Hp	Pooled heterozygosity	(14)
iHS	Integrated Haplotype Score	(15)
KLD	Kullback-Leibler divergence	(16)
LD-omega	Linkage disequilibrium based on ω statistics	(17)
meta_SS	Meta-analysis of selection signals	(18)
nSL	Number of segregating sites by length	(19)
PBS	Population branch statistic	(20)
pcadapt	Principal component analysis adaptation test	(21)
Pi	Nucleotide diversity	(22)
Pi_ratio	Nucleotide diversity ratio between two populations	(22)
ROH	Runs of homozygosity	(23)
Rsb	Cross-population integrated haplotype score (iES)	(24)
SWAD	Sliding window average difference in allele frequency	(25)
Tajima’s D	Tajima’s D statistic	(26)
XP-CLR	Cross-population composite likelihood ratios	(27)
XP-EHH	Cross-population extended haplotype homozygosity	(28)
ZHp	Z-transformed pooled heterozygosity	(14)

*Citation numbers refer to the reference list at the end of this supplementary file.

Table S2. Inclusion and exclusion criteria for selected studies

Inclusion Criteria	Exclusion Criteria
• Domesticated cattle (*Bos primigenius* lineage)<br>• All breed class (taurine, indicine, admixed and crossbred and sanga)<br>• Different breeding purposes (milk, meat, draught, fighting or dual purposes)<br>• Breeds/populations of different origins.<br>• Signatures of either positive, balancing or negative selection (selective sweeps)<br>• Statistical approach of any category.<br>• Studies that report genomic regions in a specific breed/population based on single nucleotide polymorphisms (SNPs)<br>• Selective sweeps that exceed the test threshold reported by the study<br>• Selective sweeps revealed to be linked with production, reproduction, adaptation and/or behavioral traits<br>• Studies based on UMD3.1 and ARS-UCD1.2 reference assemblies<br>• Studies that used either whole genome sequencing or any SNP genotyping technologies.<br>• Studies that used new or publicly available data<br>• In case of cross-population approaches, target breed may be compared to one or more than one population of any bovine species.<br>• Comparison might also be made based on traits, e.g., heat tolerant vs cold adapted, high vs low milk production	• Other bovine species or outgroups (e.g., Yak, Bali, Guar, Buffalo) if considered as main target population<br>• Non-peer reviewed article published in one of Beall's lists of predatory journals<br>• Reviews and conference papers<br>• No information about reference assembly and unclear methodology<br>• Analysis limited on specific chromosomes or genomic region<br>• Studies based on Btau_3.1-Btau_5.0.1 assemblies<br>• Regions represented by one position of leading SNPs

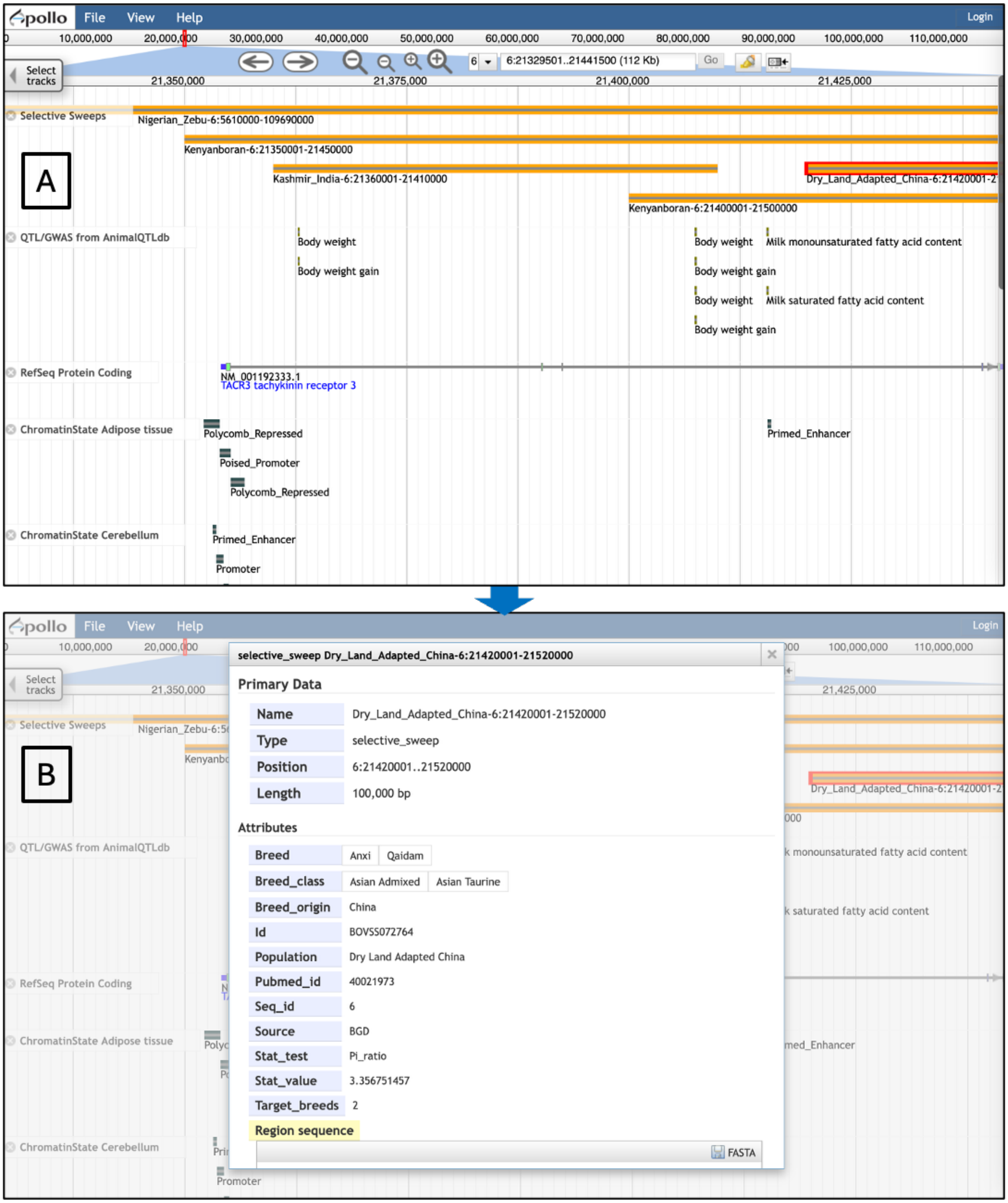

Figure S1. A) JBrowse view showing selective sweeps, QTL, RefSeq protein coding genes, and chromatin states. B) The Detail panel for the ‘Dry_Land_Adapted_China-6:21420001-21520000’ selective sweep which is viewed by right-clicking the feature.

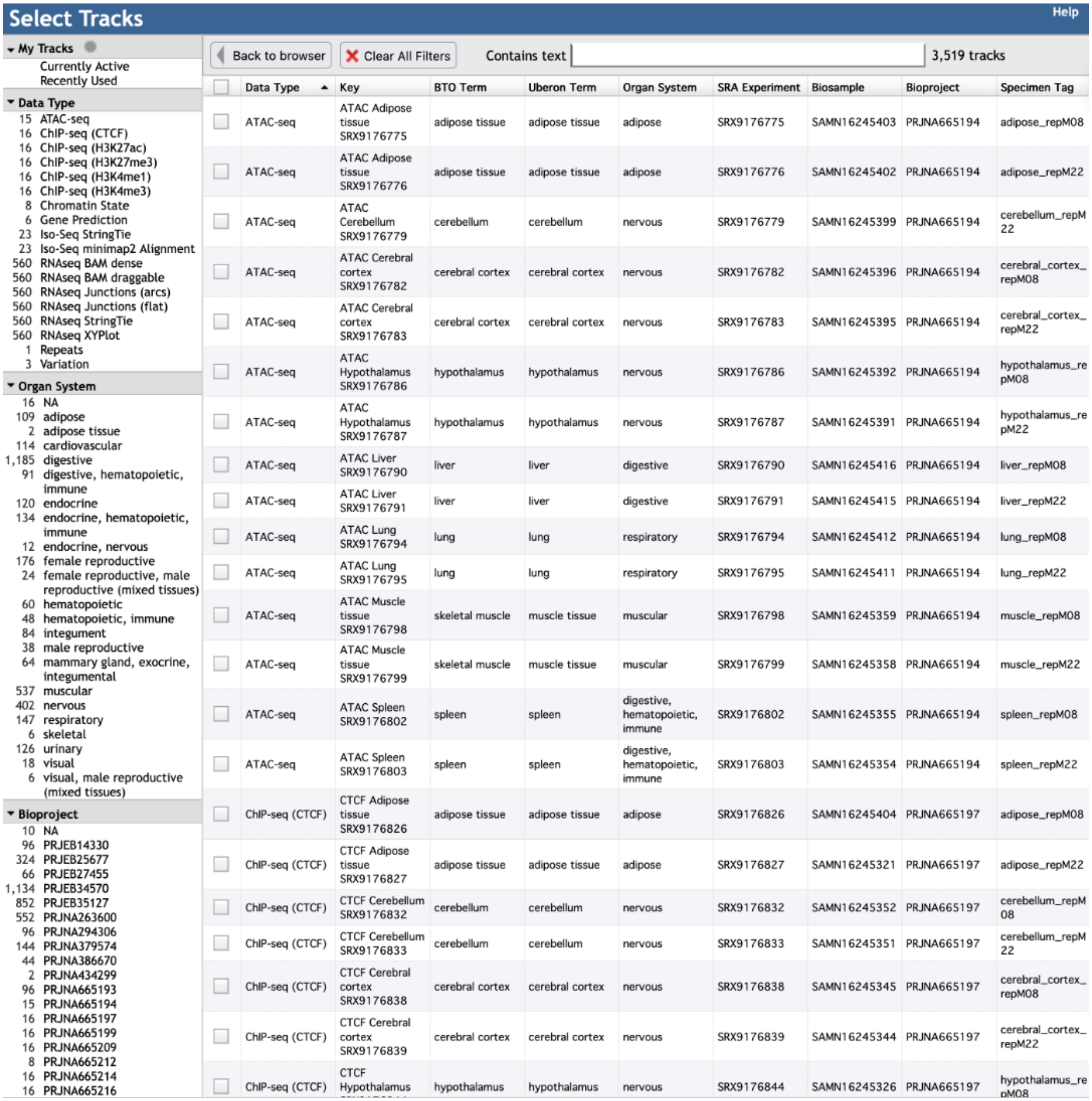

Figure S2. JBrowse faceted track selector. The selector on the left shows categories that can be selected and numbers of tracks within each category. The tracks can also be searched using the text box above the table. Selective sweeps, QTL and variants are available under the Variation category.

Example 1. Identifying Genes Within Selective Sweeps

In this example, we identify genes within selective sweeps of the population "Highland Bolivian Creole" and then perform enrichment analysis of the gene list to identify overrepresented Gene Ontology (GO) and pathway terms. First, go to the Query Template Bar in the middle of the BovineMine homepage, and select Variation. Template queries are listed. If you do not see the appropriate query, click 'More queries' to see the full list. For this example, select 'Population → Selective Sweeps and Genes".

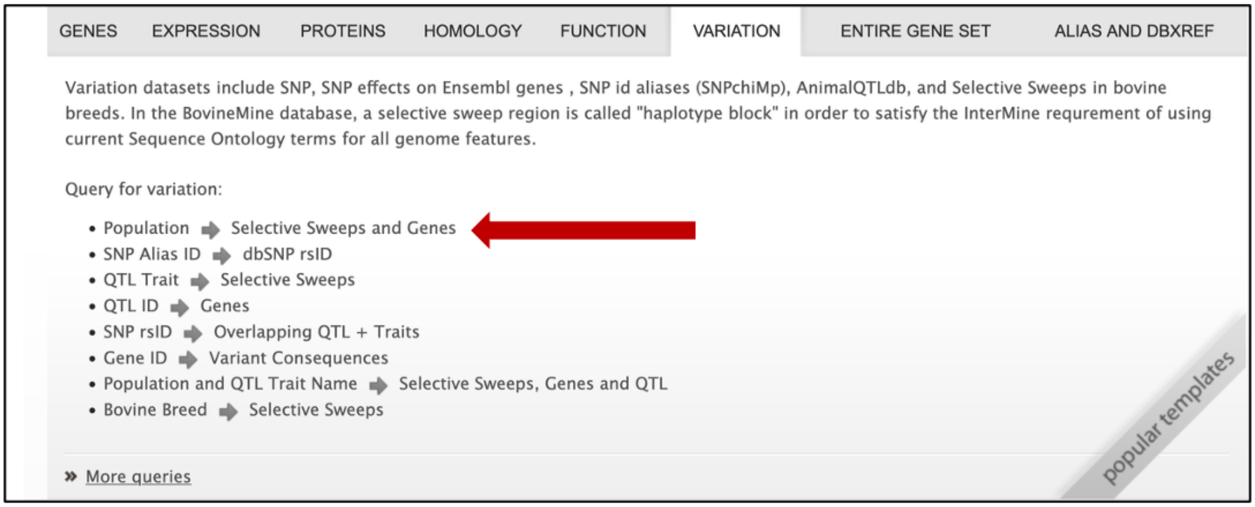

Figure S3. The Template Bar on the BovineMine home page showing the Variation Tab.

Clicking the query name opens a menu. Select 'Highland Bolivian Creole' as the population, then click 'Show Results'.

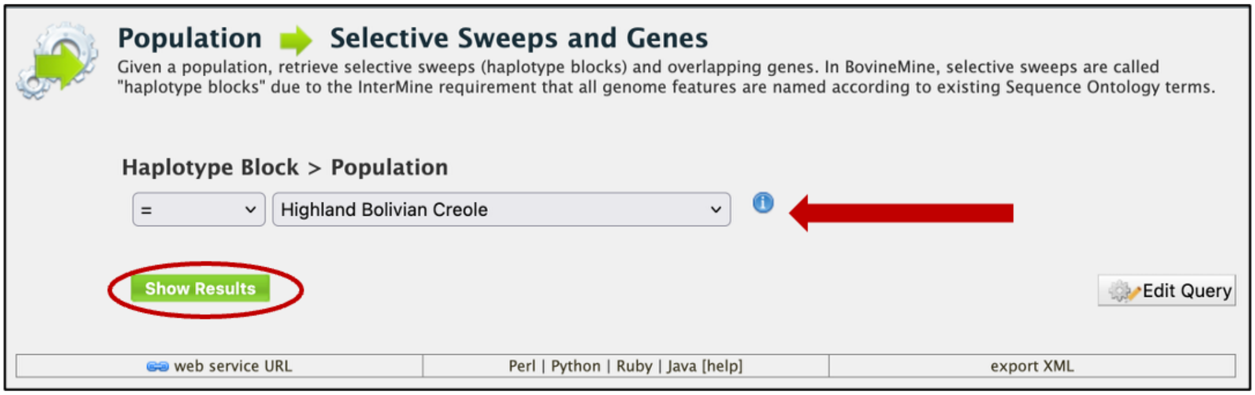

Figure S4. The 'Population → Selective Sweeps and Genes' template query menu.

The query output is provided as a table that can be modified by deleting and hiding columns, and sorting and filtering rows using icons above the columns. Additional columns can be added with ‘Manage Columns’. The Export button above the table allows downloading in various formats.

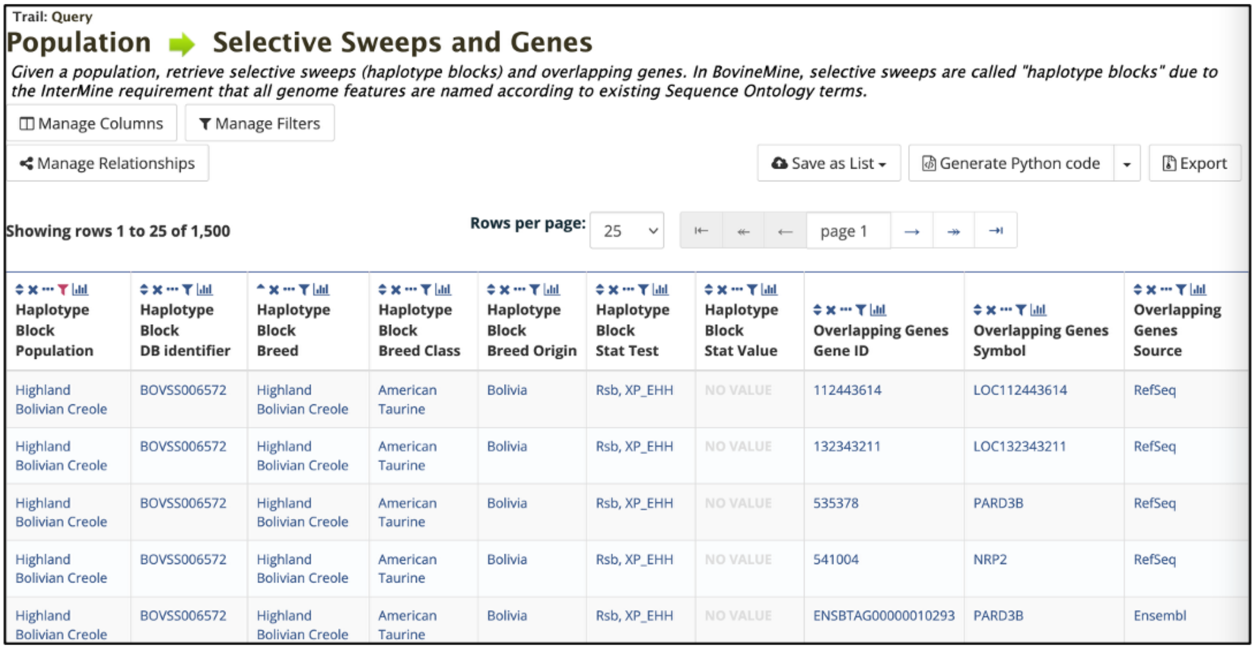

Figure S5. Output from query performed in Figure S4.

We will save gene lists individually for RefSeq and Ensembl genes, because enrichment analysis must be performed with only one gene set at a time for statistics to be valid. Use the histogram icon above the ‘Overlapping Genes Source’ column to see the gene sources (Ensembl or RefSeq), and select ‘RefSeq’ and ‘Restrict table to matching rows’ so that a list of RefSeq genes can be saved.

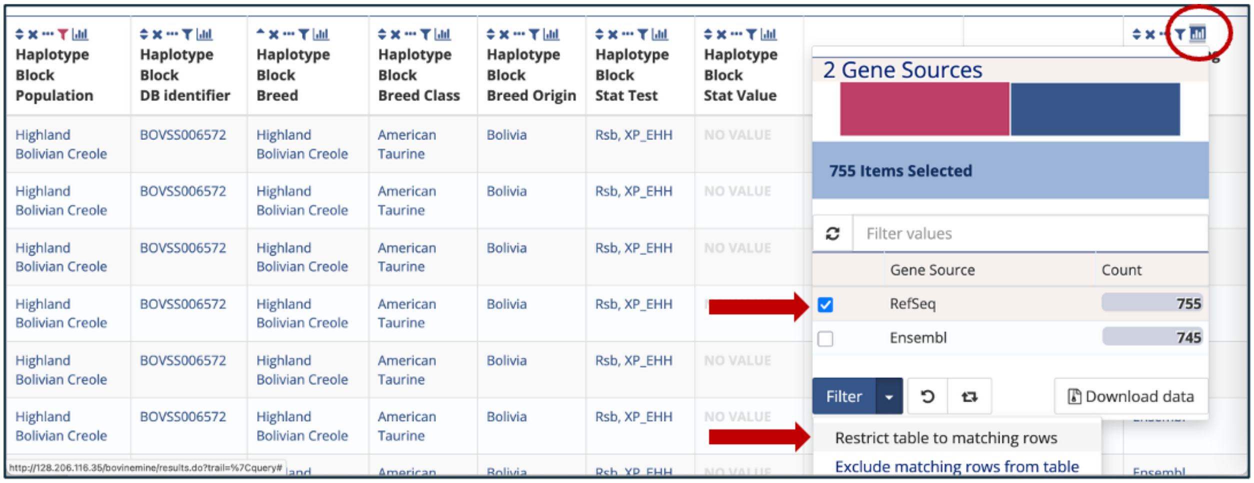

Figure S6. Filtering the table for only RefSeq genes.

After filtering for RefSeq, click ‘Save as a List’, then click ‘Haplotype Block > Overlapping Genes (672 Genes)’. A menu will open allowing you to enter a name for the list. After entering a name, click ‘Create list’. Wait a few moments, and once the menu has disappeared the list has been saved. Then use the ‘Undo’ button above the table to undo the filtering for RefSeq genes. This brings back the Ensembl genes, and you can repeat the filtering and the process of saving a list for the Ensembl genes.

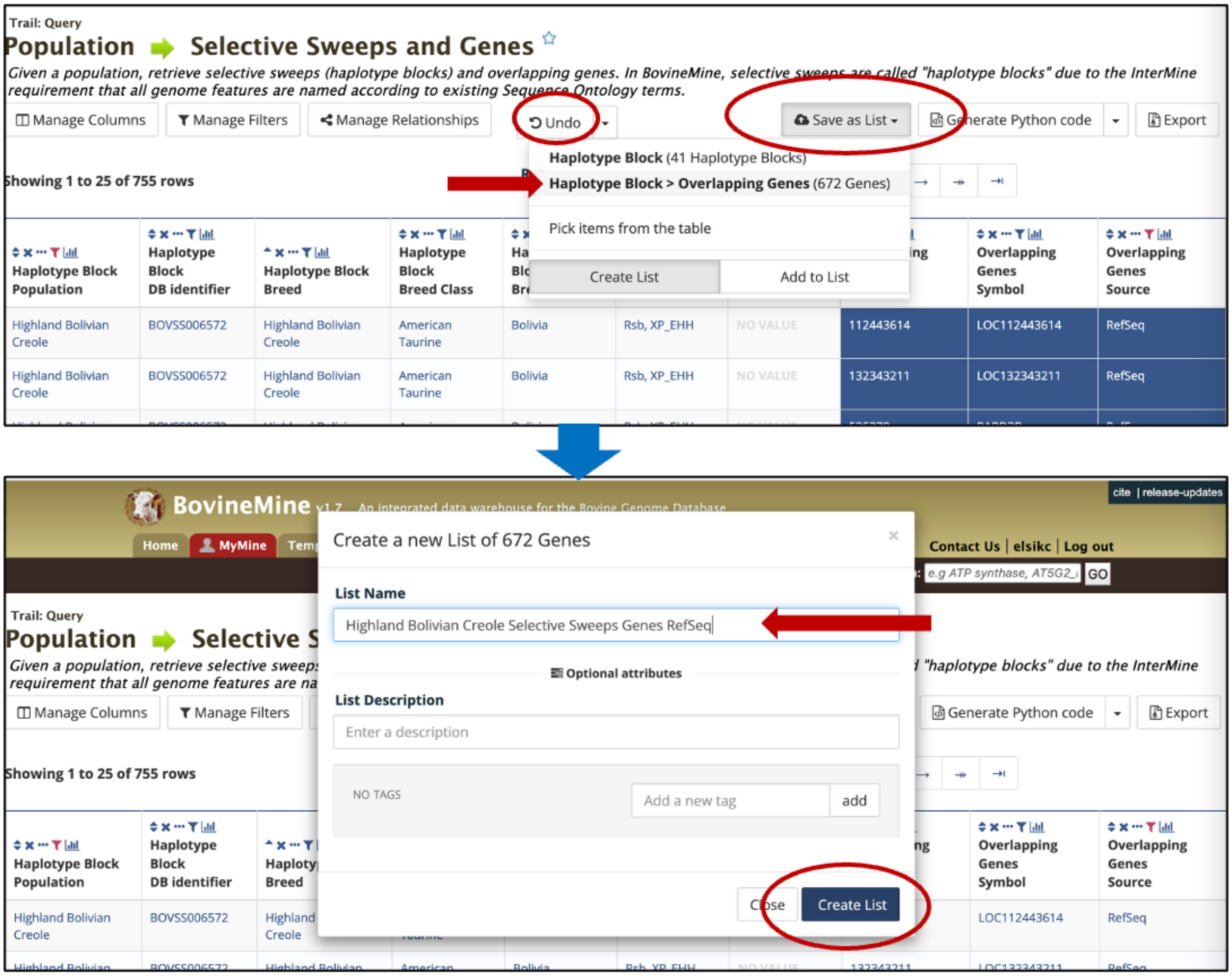

Figure S7. Filtering for RefSeq genes and then saving them as a list.

The lists are available by clicking the List Tab in the main BovineMine navigation bar, then toggling to 'View'. Click on a list name to go to the List Analysis page.

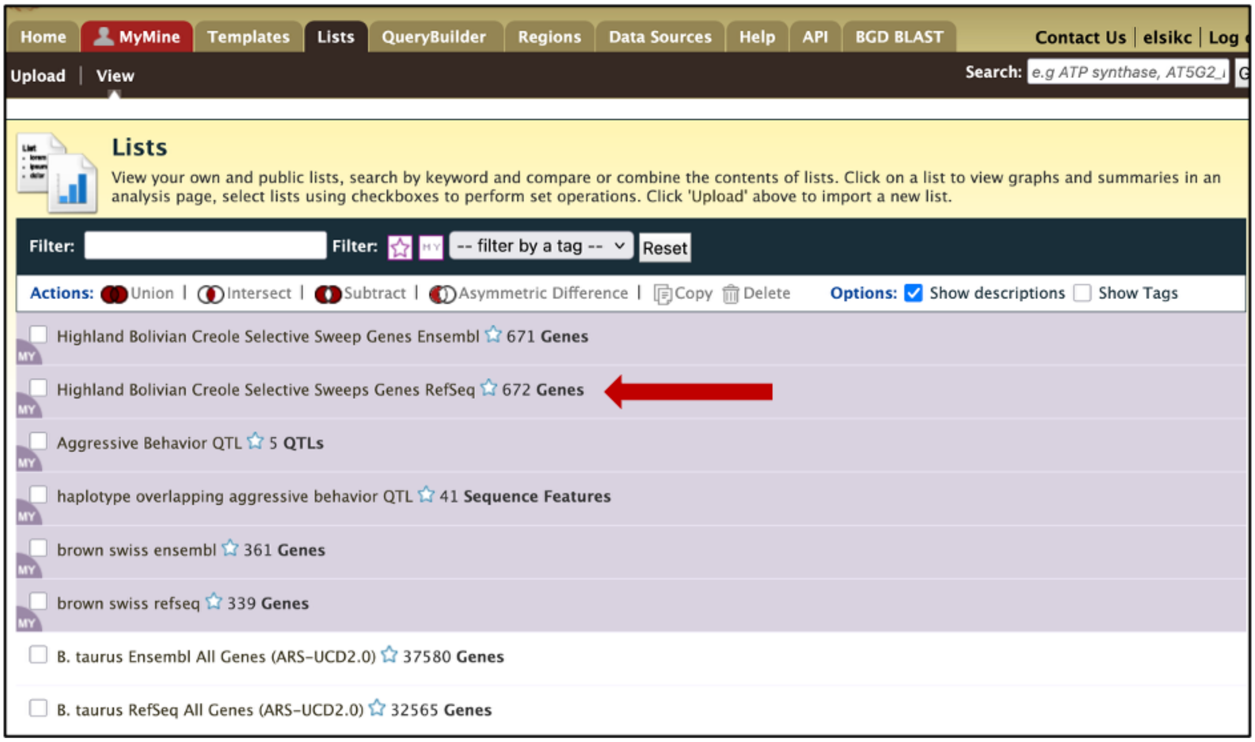

Figure S8. List View page. Lists created by the user are shown in a mauve background. Lists in a white background are available to all users by default.

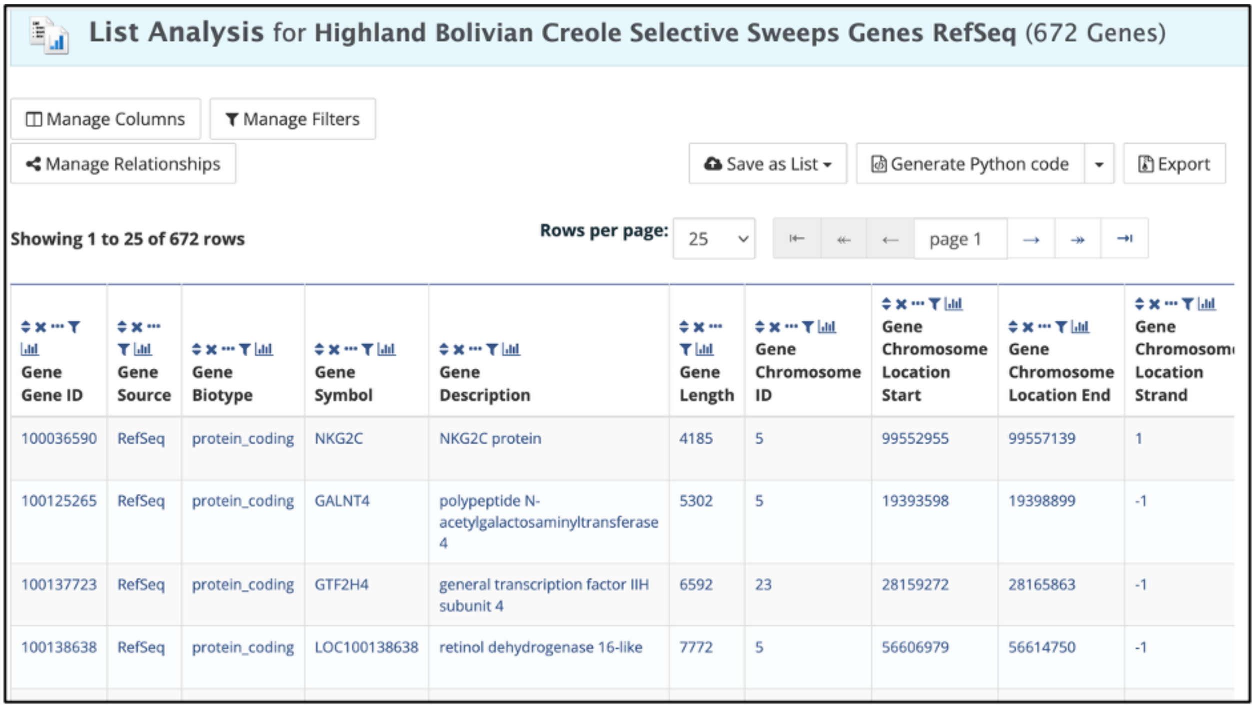

Figure S9. List Analysis Page.

Below the table in the List Analysis page are Gene Ontology and Pathway Enrichment widgets, which are activated automatically. For proper statistics, you must use the correct background gene set (‘Backgound population’). Change the background gene set for each widget separately using the ‘Change’ button below ‘Background population’. All saved gene lists, including the entire bovine RefSeq and Ensembl gene sets are available in the pulldown menu. Select ‘B. taurus RefSeq All Genes’. You can also change the Test Correction method and the P-value threshold. For GO enrichment analysis, you can select the ontology (Biological Process, Molecular Function or Cellular Component). For pathway enrichment analysis, you can select the Pathway dataset. Due to the gene set used by the external resources, KEGG is available for RefSeq but not Ensembl, and Reactome is available for Ensembl but not RefSeq. You can download tab-delimited files of the enrichment results for each analysis by clicking ‘Download’.

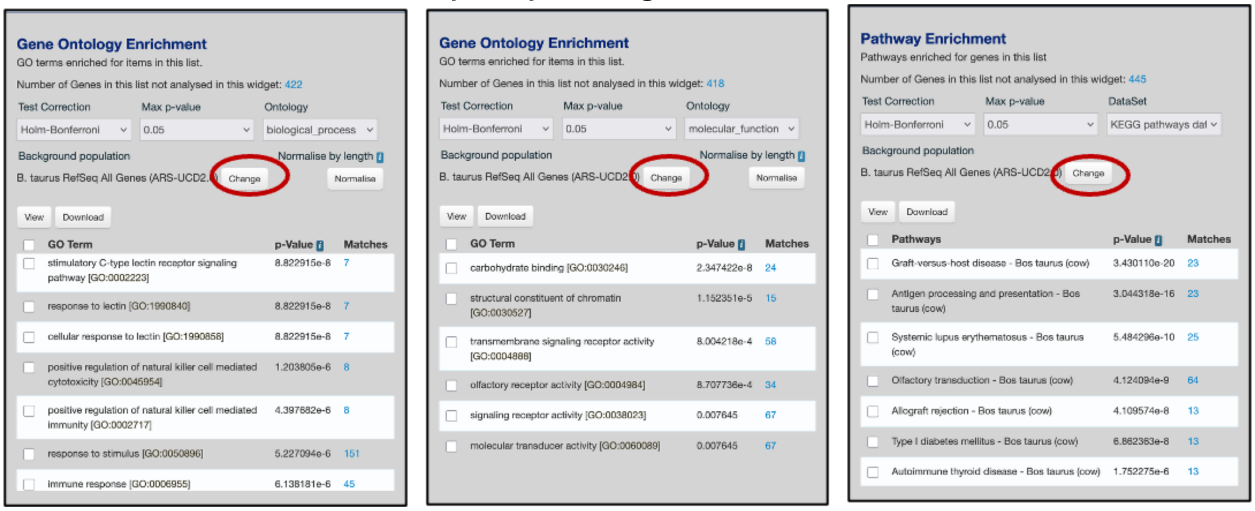

Figure S10. Enrichment analyses for the saved RefSeq gene list.

You can repeat the process for the Ensembl gene list by going back to the List View page and clicking on your saved Ensembl gene list.

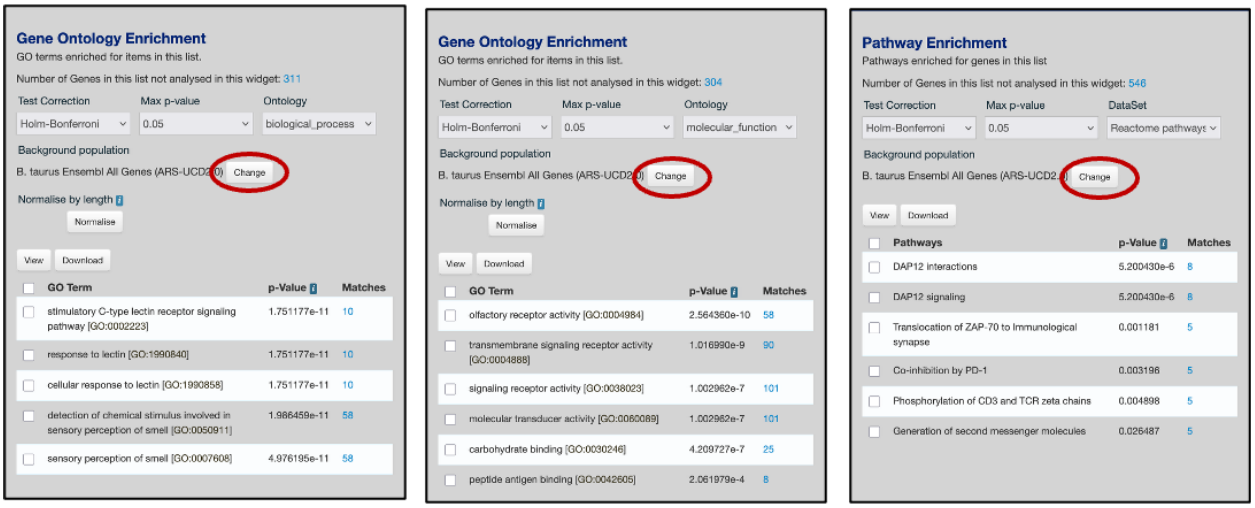

Figure S11. Enrichment analyses for the saved Ensembl gene list.

Example 2. Identifying QTL Within Selective Sweeps, and Performing Enrichment Analysis of Genes within Selected Sweeps Harboring QTL

In this example we identify QTL that overlap selective sweeps of the Finnish Ayrshire population, then we filter for sweep regions with milk fat QTL, retrieve genes from those regions, and perform enrichment analysis to identify overrepresented GO and pathway terms for those genes. First select the 'Population → Selective Sweeps and QTL' template query, available under the 'Variation' tab on the BovineMine home page. Select 'Finnish Ayrshire Finland' as the population, and then click 'Show Results'

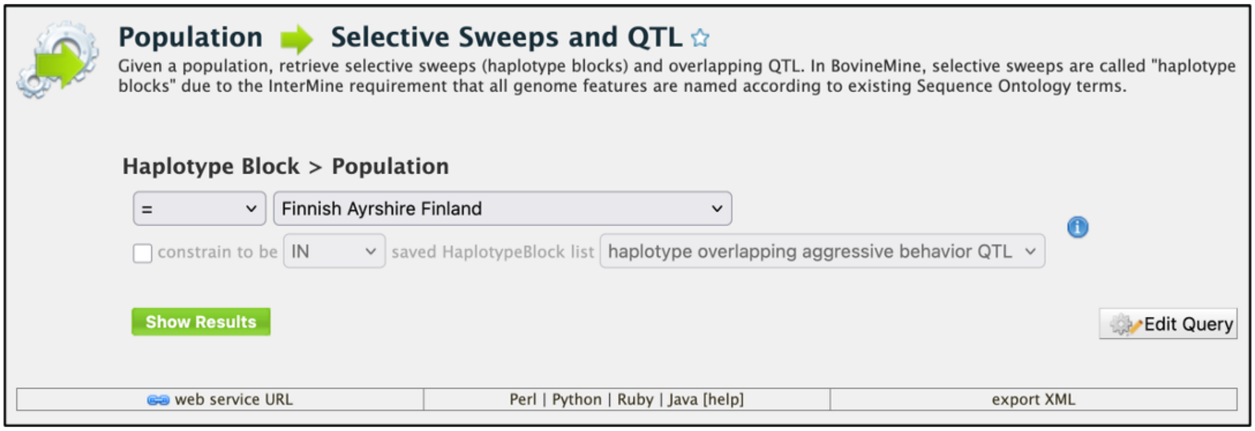

Figure S12. 'Population → Selective Sweeps and QTL' template query menu.

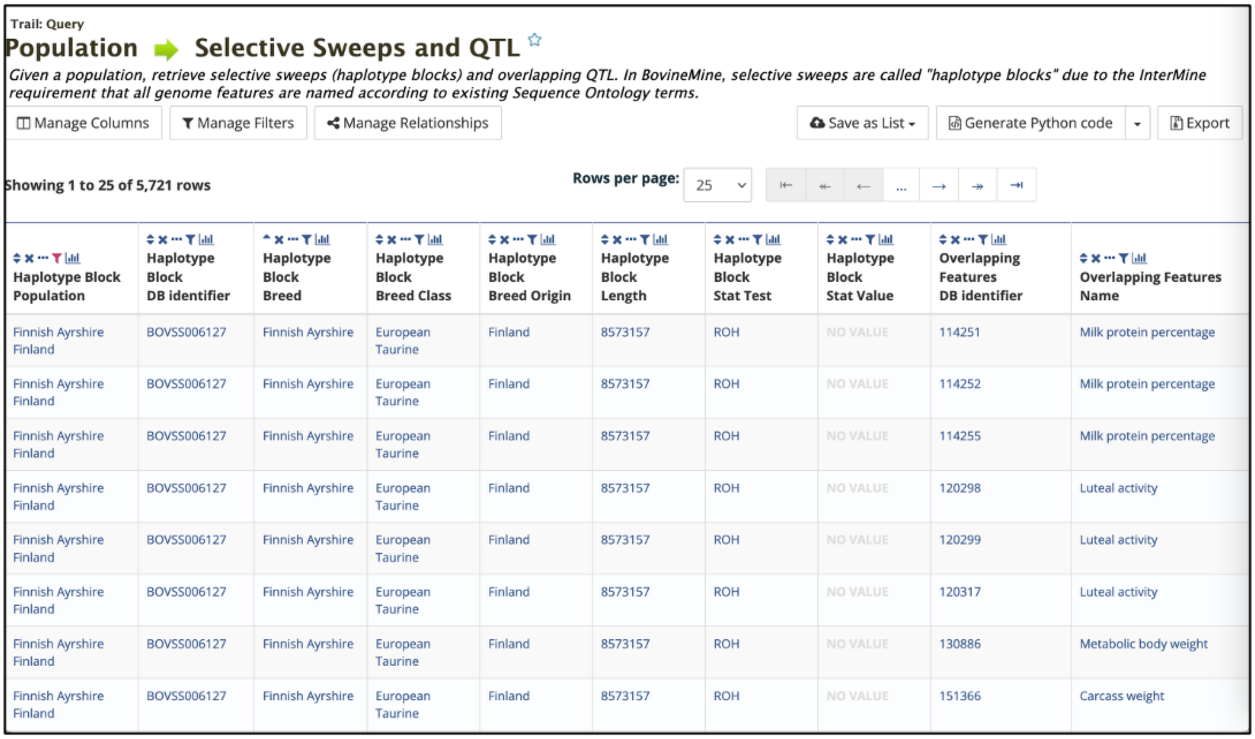

Haplotype Block Population	Haplotype Block DB identifier	Haplotype Block Breed	Haplotype Block Breed Class	Haplotype Block Breed Origin	Haplotype Block Length	Haplotype Block Stat Test	Haplotype Block Stat Value	Overlapping Features DB identifier	Overlapping Features Name
Finnish Ayrshire Finland	BOVSS006127	Finnish Ayrshire	European Taurine	Finland	8573157	ROH	NO VALUE	114251	Milk protein percentage
Finnish Ayrshire Finland	BOVSS006127	Finnish Ayrshire	European Taurine	Finland	8573157	ROH	NO VALUE	114252	Milk protein percentage
Finnish Ayrshire Finland	BOVSS006127	Finnish Ayrshire	European Taurine	Finland	8573157	ROH	NO VALUE	114255	Milk protein percentage
Finnish Ayrshire Finland	BOVSS006127	Finnish Ayrshire	European Taurine	Finland	8573157	ROH	NO VALUE	120298	Luteal activity
Finnish Ayrshire Finland	BOVSS006127	Finnish Ayrshire	European Taurine	Finland	8573157	ROH	NO VALUE	120299	Luteal activity
Finnish Ayrshire Finland	BOVSS006127	Finnish Ayrshire	European Taurine	Finland	8573157	ROH	NO VALUE	120317	Luteal activity
Finnish Ayrshire Finland	BOVSS006127	Finnish Ayrshire	European Taurine	Finland	8573157	ROH	NO VALUE	130886	Metabolic body weight
Finnish Ayrshire Finland	BOVSS006127	Finnish Ayrshire	European Taurine	Finland	8573157	ROH	NO VALUE	151366	Carcass weight

Figure S13. Output from query performed in Figure S12.

Click the histogram icon above the ‘Overlapping Features Name’ column to see the numbers of QTL traits. Clicking ‘Download data’ in the resulting panel would allow you to save a tab delimited file of the QTL traits and counts. However, instead we use the menu to filter the table to show only rows for ‘Milk fat yield’, so that we can save a list of selective sweeps with milk fat yield QTL.

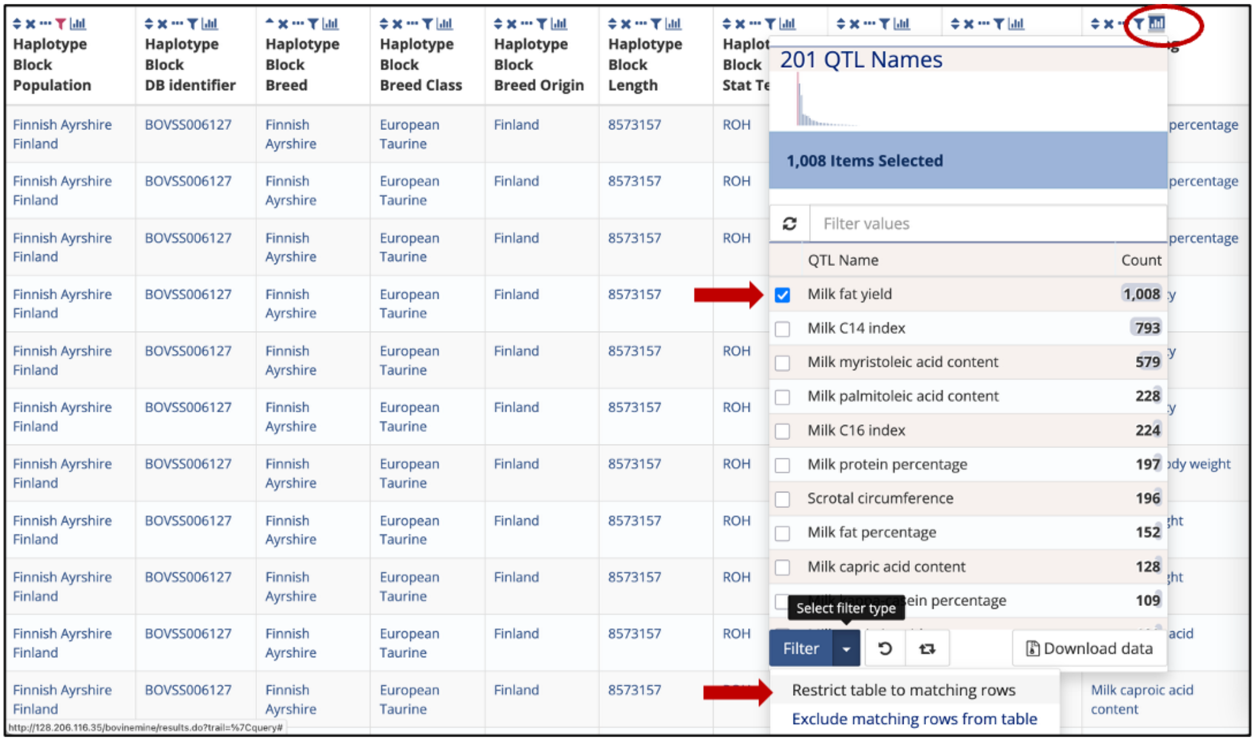

Figure S14. Filtering the output for Milk fat yield.

In the filtered table, click the histogram above the ‘Haplotype Block DB Identifier’ to see how many selective sweeps (referred to as ‘haplotype blocks’ in BovineMine) contain milk fat yield QTL, and the distribution of QTL among these regions. You can see that most of the QTL are in a single region.

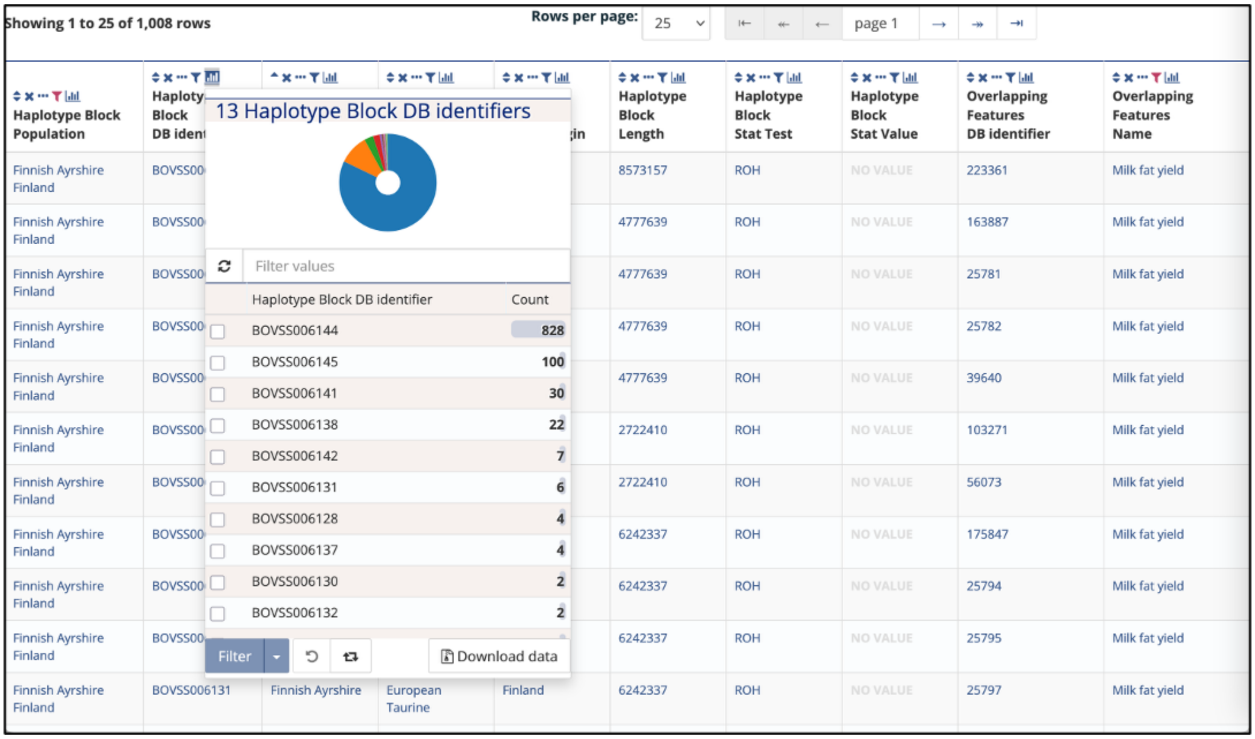

Figure S15. Viewing the number rows for the 13 selective sweeps that contain milk fat QTL.

The next step is to save a list of the selective sweeps (referred to as Haplotype Blocks in BovineMine) using steps like those described in Example 1 for saving gene lists.

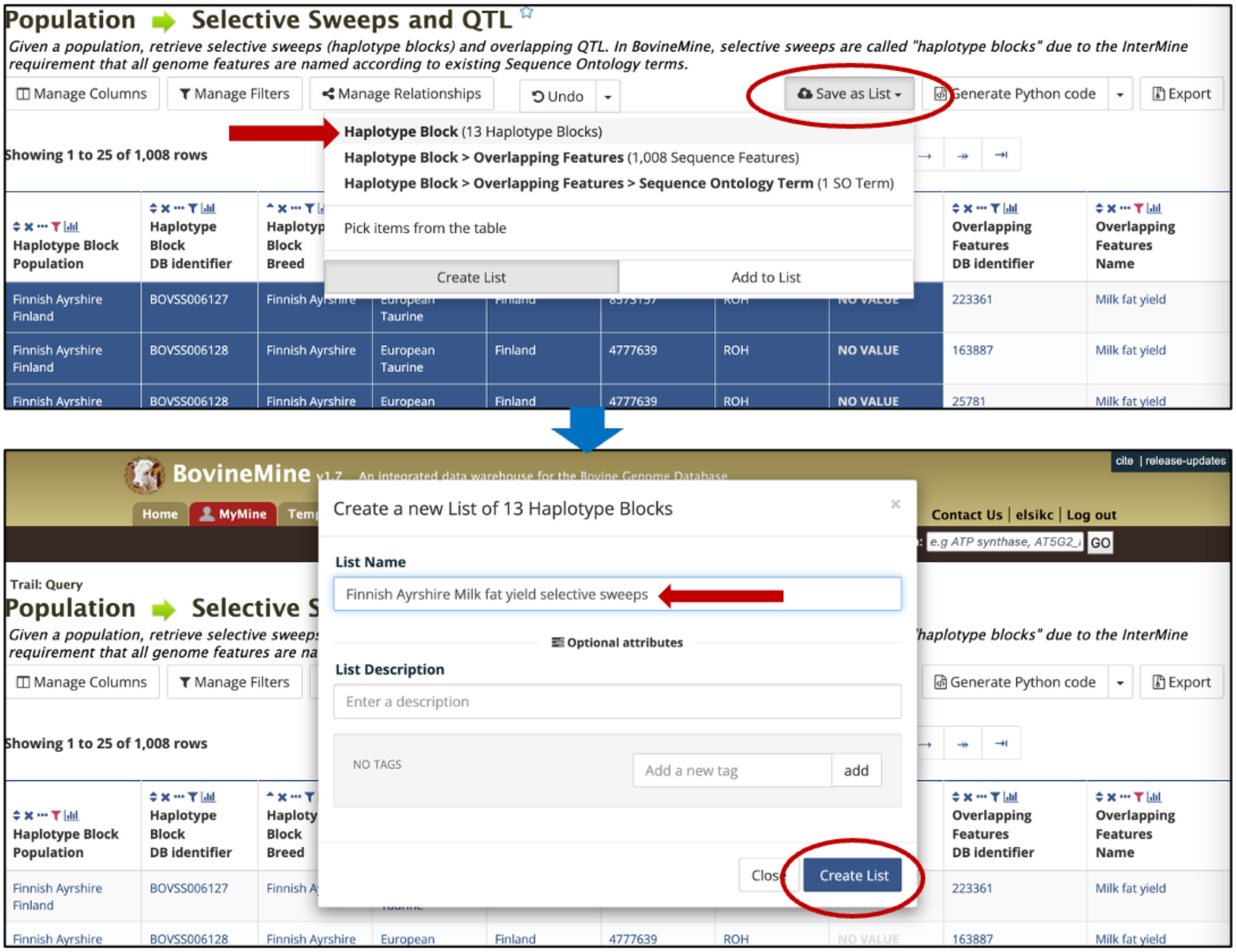

Figure S16. Save a list of selective sweeps containing milk fat QTL.

Perform a query for genes within selective sweep regions that overlap QTL using the 'Selective Sweep (Haplotype Block) ID → Genes' template query, available under the Variation tab on the BovineMine home page. Check the box to the left of "constrain to be". This will allow you to use a pulldown menu to select one of your lists, so that the query will be run on the entire list of IDs. Select the list you saved in Figure S16. We called our list 'Finnish Ayshire Milk fat yield sweeps'.

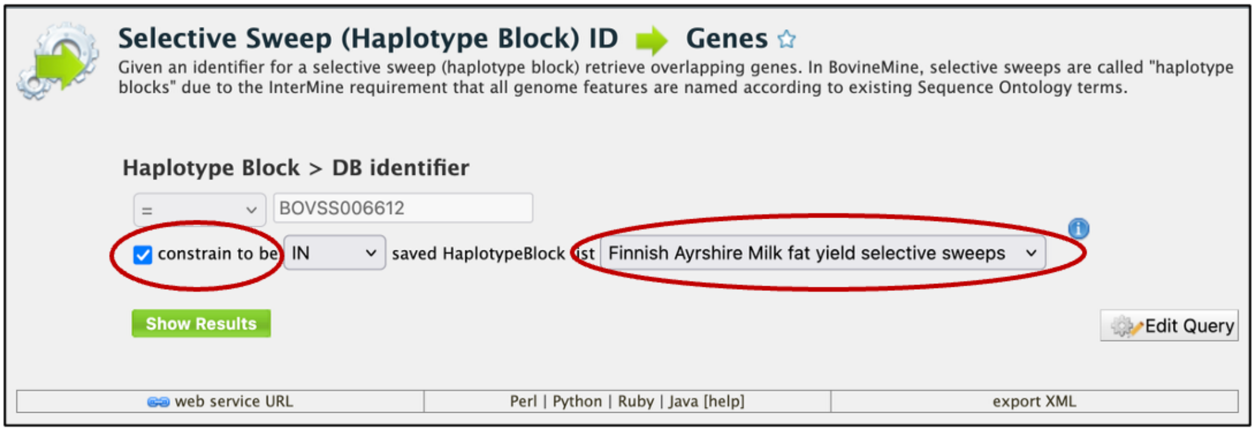

Figure S17. 'Selective Sweep (Haplotype Block) ID → Genes' template query menu, showing that a list is selected as the query constraint.

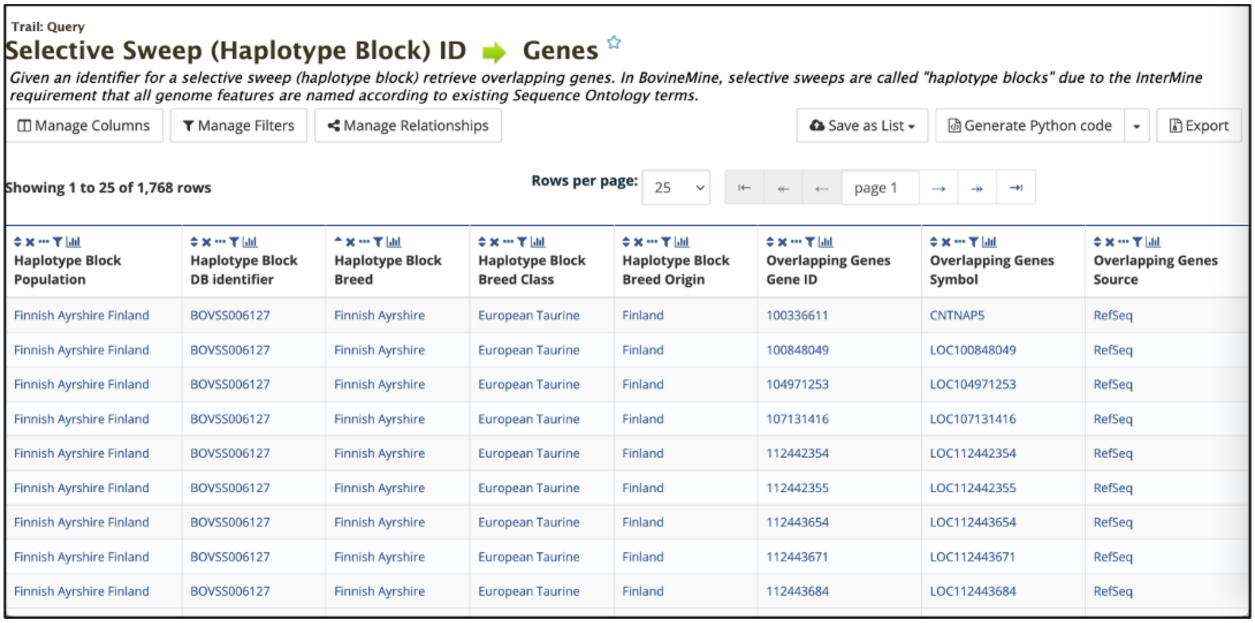

Haplotype Block Population	Haplotype Block DB identifier	Haplotype Block Breed	Haplotype Block Breed Class	Haplotype Block Breed Origin	Overlapping Genes Gene ID	Overlapping Genes Symbol	Overlapping Genes Source
Finnish Ayrshire Finland	BOVSS006127	Finnish Ayrshire	European Taurine	Finland	100336611	CNTNAP5	RefSeq
Finnish Ayrshire Finland	BOVSS006127	Finnish Ayrshire	European Taurine	Finland	100848049	LOC100848049	RefSeq
Finnish Ayrshire Finland	BOVSS006127	Finnish Ayrshire	European Taurine	Finland	104971253	LOC104971253	RefSeq
Finnish Ayrshire Finland	BOVSS006127	Finnish Ayrshire	European Taurine	Finland	107131416	LOC107131416	RefSeq
Finnish Ayrshire Finland	BOVSS006127	Finnish Ayrshire	European Taurine	Finland	112442354	LOC112442354	RefSeq
Finnish Ayrshire Finland	BOVSS006127	Finnish Ayrshire	European Taurine	Finland	112442355	LOC112442355	RefSeq
Finnish Ayrshire Finland	BOVSS006127	Finnish Ayrshire	European Taurine	Finland	112443654	LOC112443654	RefSeq
Finnish Ayrshire Finland	BOVSS006127	Finnish Ayrshire	European Taurine	Finland	112443671	LOC112443671	RefSeq
Finnish Ayrshire Finland	BOVSS006127	Finnish Ayrshire	European Taurine	Finland	112443684	LOC112443684	RefSeq

Figure S18. Output of the query performed in Figure S17.

You can now filter and save individual RefSeq and Ensembl gene lists, then view the List Analysis pages and the enrichment widgets, as described in Example 1 (Figures S5-S8).

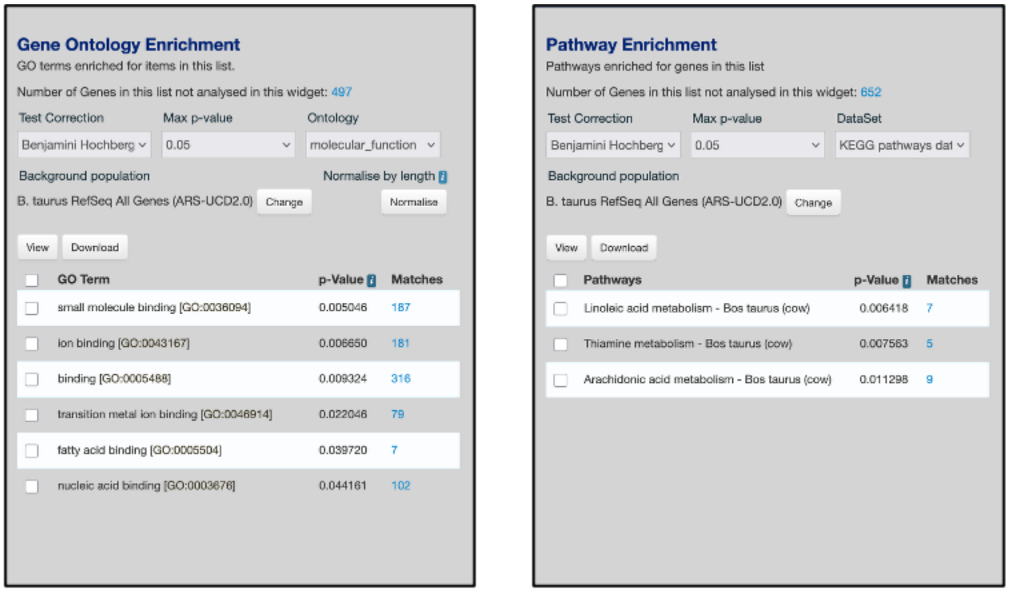

Figure S19. Gene Ontology (molecular function) and KEGG pathway enrichment for RefSeq genes within Finnish Ayrshire selective sweeps overlapping milk fat QTL. There were no significant terms for biological process.

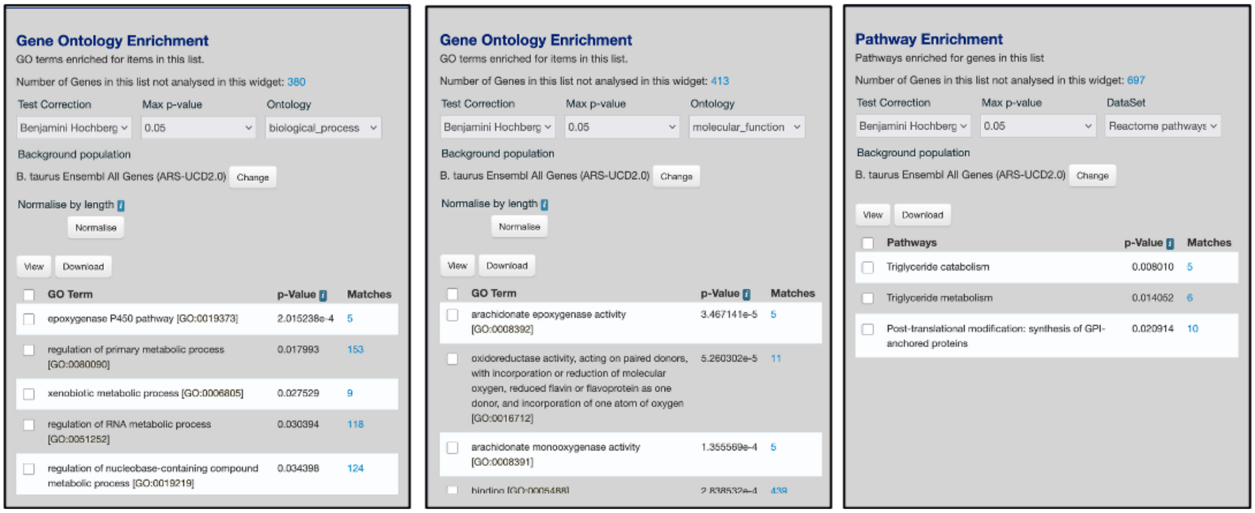

Figure S20. Gene Ontology and Reactome pathway enrichment for Ensembl genes within Finnish Ayrshire selective sweeps overlapping milk fat QTL.

Example 3. Identifying Populations and Breeds with Selective Sweeps Harboring QTL for a Specified Trait

In this example, we identify which populations and breeds have aggressive behavior QTL within selective sweeps, using the 'QTL Trait → Selective Sweeps' query template. You can click on the histograms above the 'Haplotype Block Population' and 'Haplotype Block Breeds' columns to see the numbers of populations or breeds that contain aggressive behavior QTL within selective sweeps.

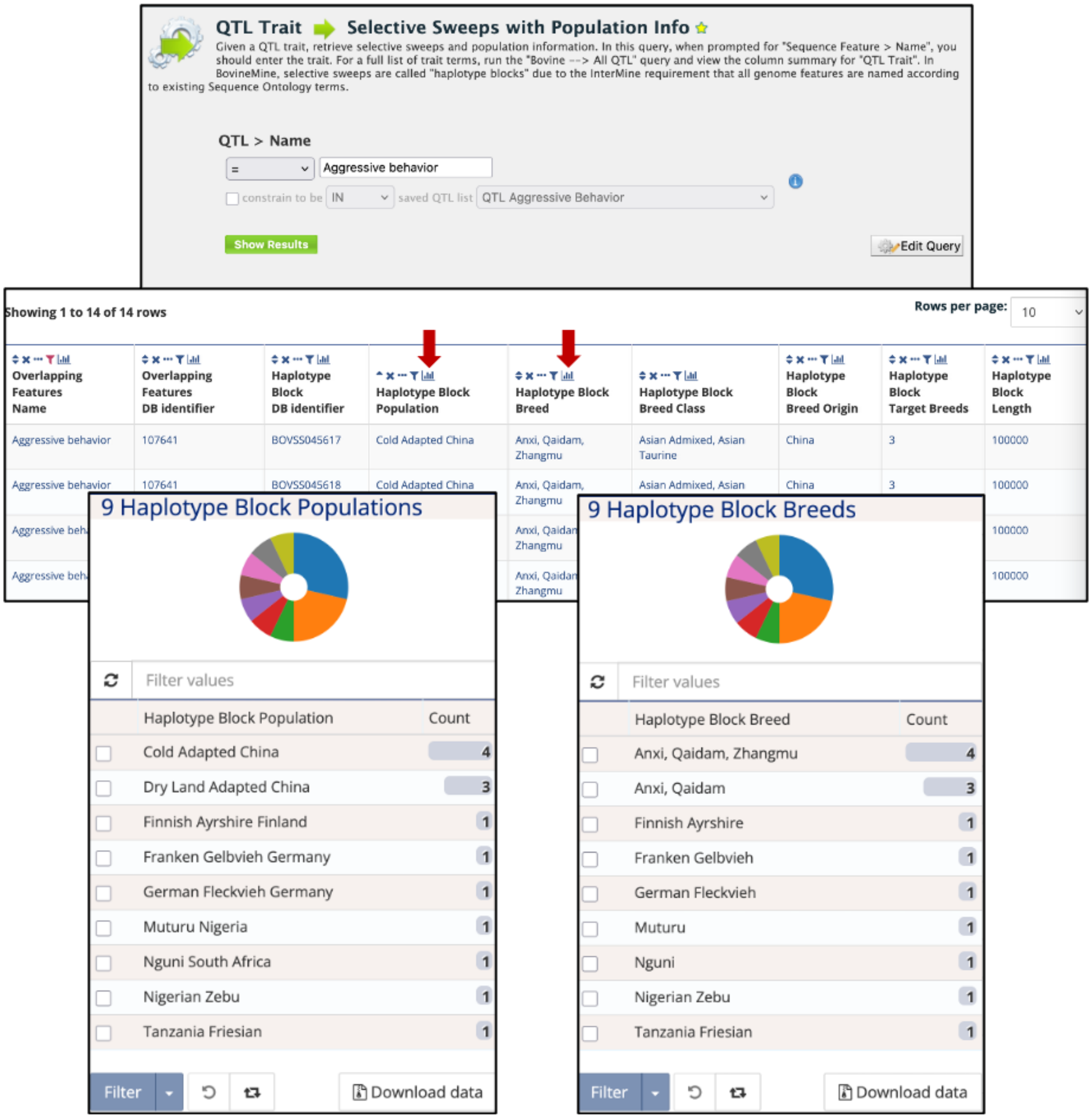

Figure S21. Identifying populations and breeds with aggressive behavior QTL within selective sweeps.

Example 4. Uploading Coordinates of Significant GWAS SNP to Regions Search to Identify Overlapping Selective Sweeps

In this example, we identify selective sweeps that overlap an uploaded list of coordinates of significant genome wide association study (GWAS) SNP for body conformation traits in Canadian Holstein cattle (29). The article is freely available: https://www.frontiersin.org/journals/genetics/articles/10.3389/fgene.2024.1478788/full

Download Supplementary Table 4 using the following link: https://www.frontiersin.org/journals/genetics/articles/10.3389/fgene.2024.1478788/full#supplementary-material

Column A contains the chromosome number and column B contains the SNP coordinate. The Regions Search in BovineMine requires both a start and end position, so copy columns A and B into a new spreadsheet tab. Then copy column B into column C, so that you will have both start and end coordinates (even though they are the same number).

Go to the Regions Search page by clicking 'Regions' in the main BovineMine navigation bar. The choices for step 1 (Select Organism) and step 2 (Select Assembly) are automatically selected because Bos taurus ARS-UCD2.0 is the only genome assembly in BovineMine. For step 3 (Select Feature Types), check the box next to 'Haplotype Block'. In the development of BovineMine, we were required to use only current Sequencing Ontology terms for all genome features, and haplotype_block was the closest available term.

The last step is to copy and paste the chromosome coordinates (chromosome number, start and end coordinate) from the Excel spreadsheet into the search box, then click 'Search' in the bottom right corner.

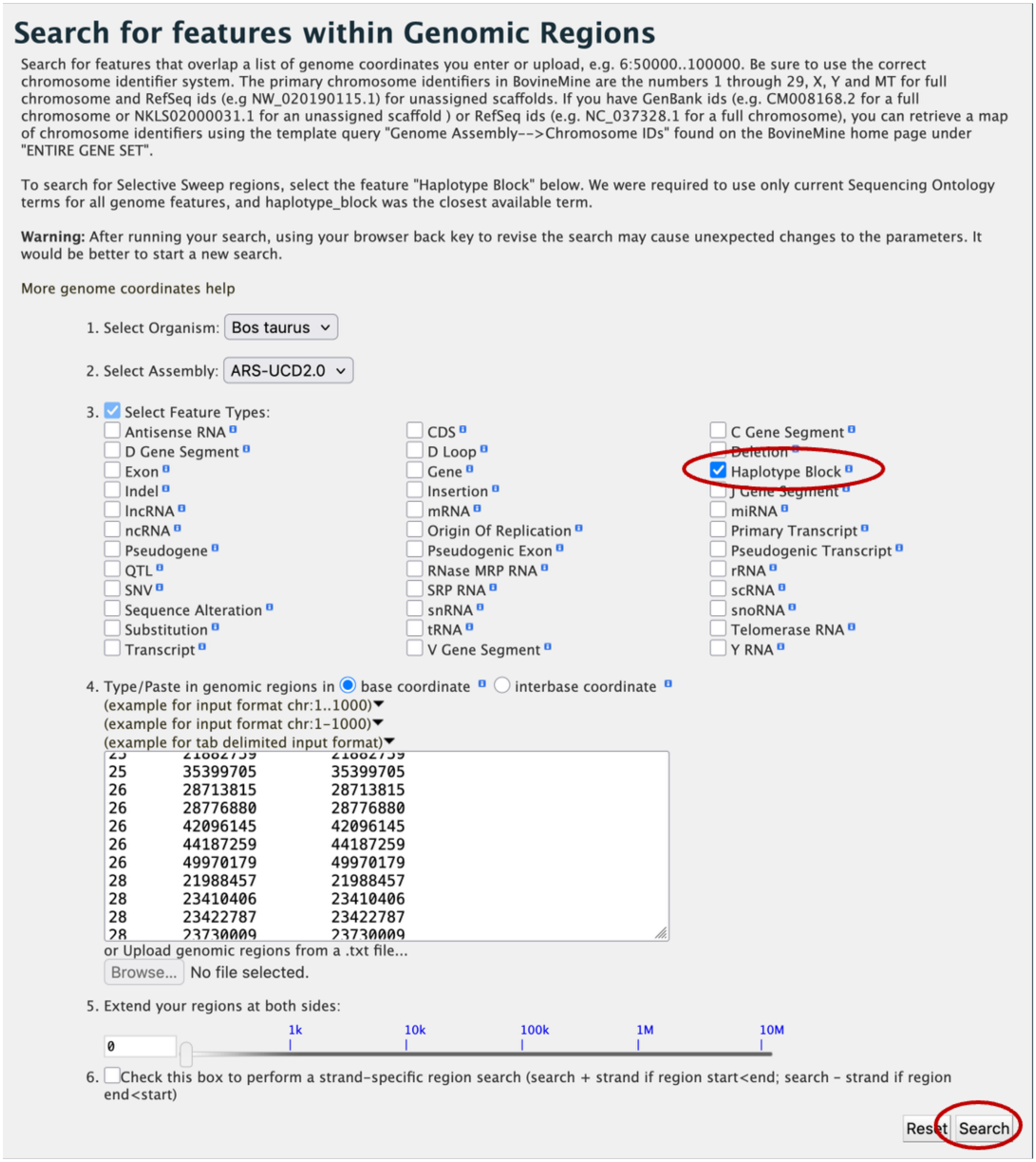

Figure S22. Regions search menu.

The Regions Search output provides results for each set of coordinates. These can be downloaded in various formats or saved as a list individually using buttons shown in the first column for each region, or they can all be downloaded or saved as a list together using buttons to the top left of the table. For this example, save the selective sweeps (referred to as Haplotype Blocks) by clicking ‘Go’ next the ‘Create list by feature type’.

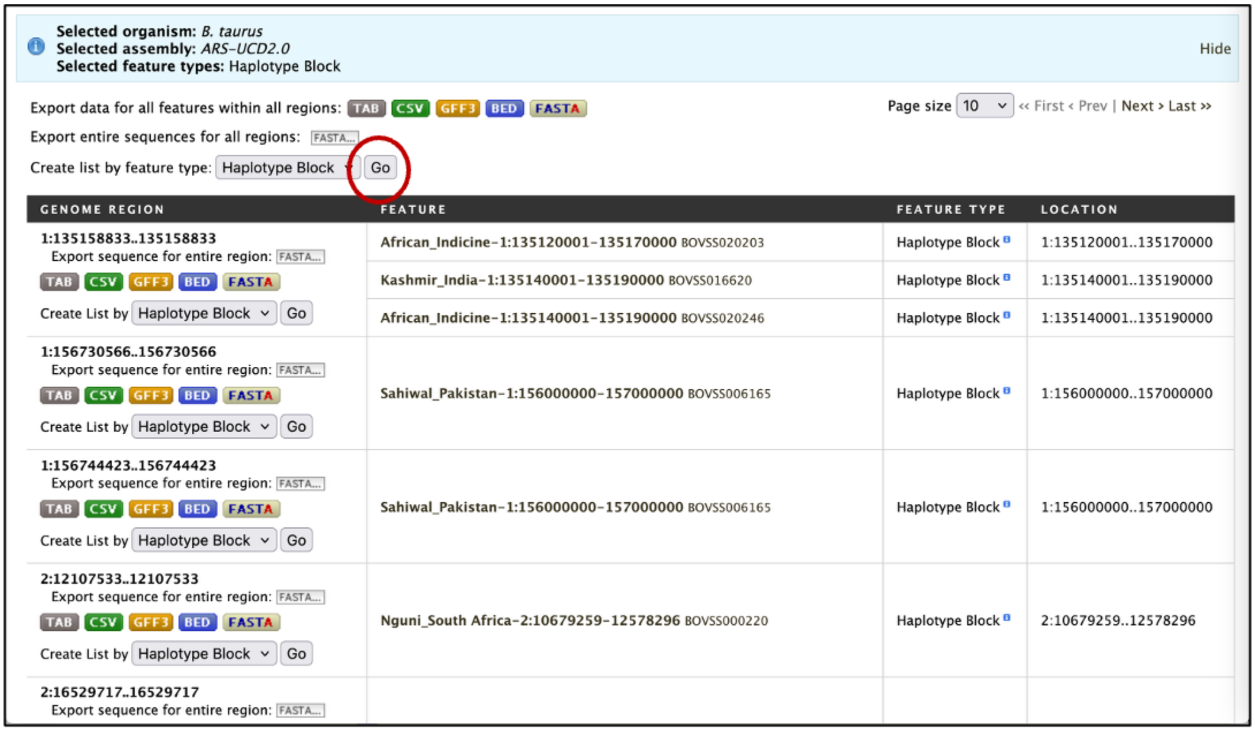

Figure S23. Regions search output page.

Clicking ‘Go’ saves the list. It also brings you to the List Analysis page showing some default information about the selective sweep regions (see Figure S22, next page). For region searches, the lists are automatically assigned names. You can change the name by toggling to your lists under the ‘MyMine’ tab.

In the table on the List Analysis page, you can click the histogram icons above the ‘Haplotype Block Population’ and ‘Haplotype Block Breed’ columns to view the population and breeds within selective sweeps overlapping the uploaded QTL. You can filter for ‘Holstein’ to view the Holstein populations and breeds with these selective sweeps.

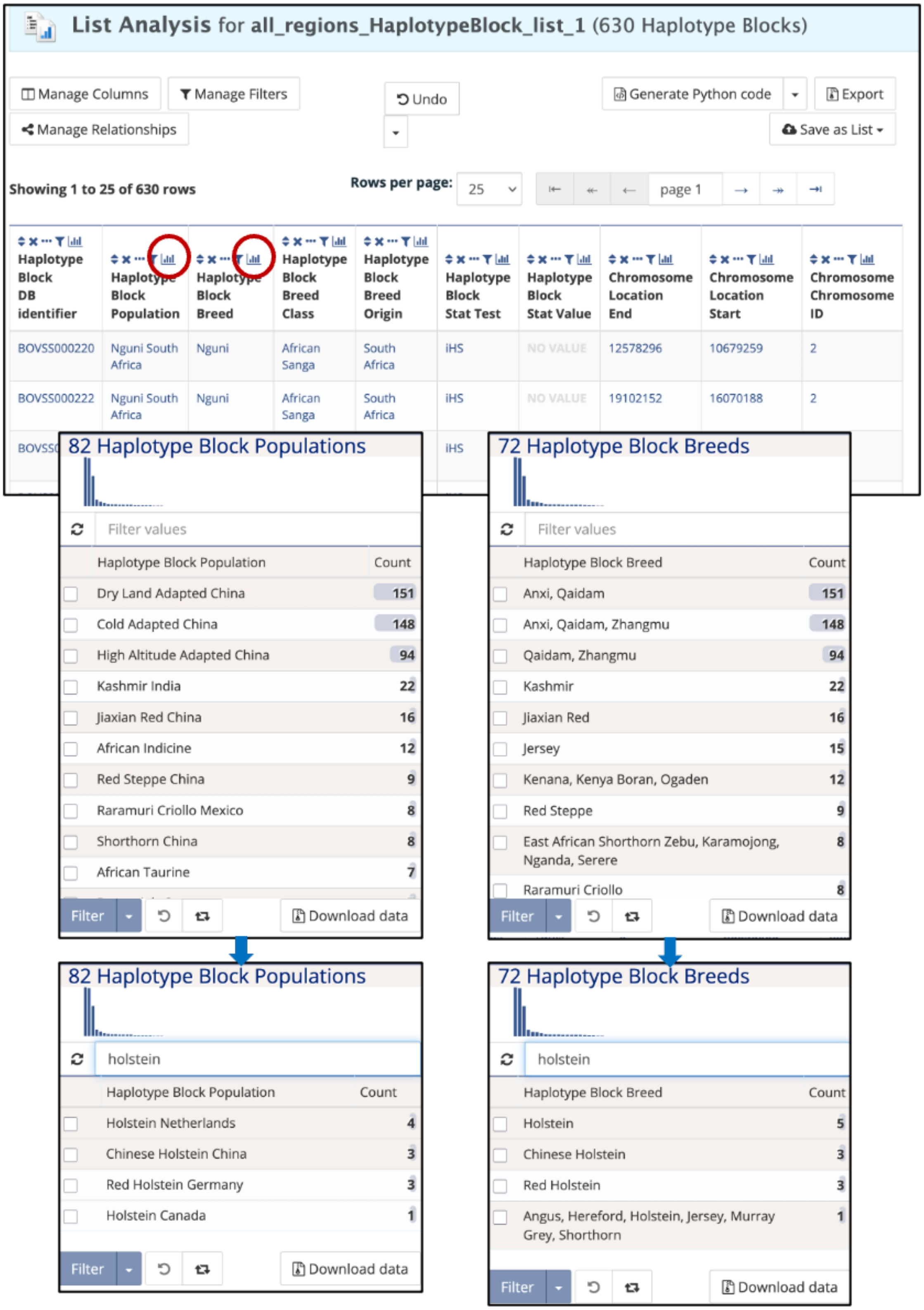

Figure S24. Viewing the populations and breeds by clicking histogram icons above the columns in the List Analysis table in the List Analysis page, and filtering each list with for terms containing ‘Holstein’.

Filter the table to show only rows for Holstein breeds.

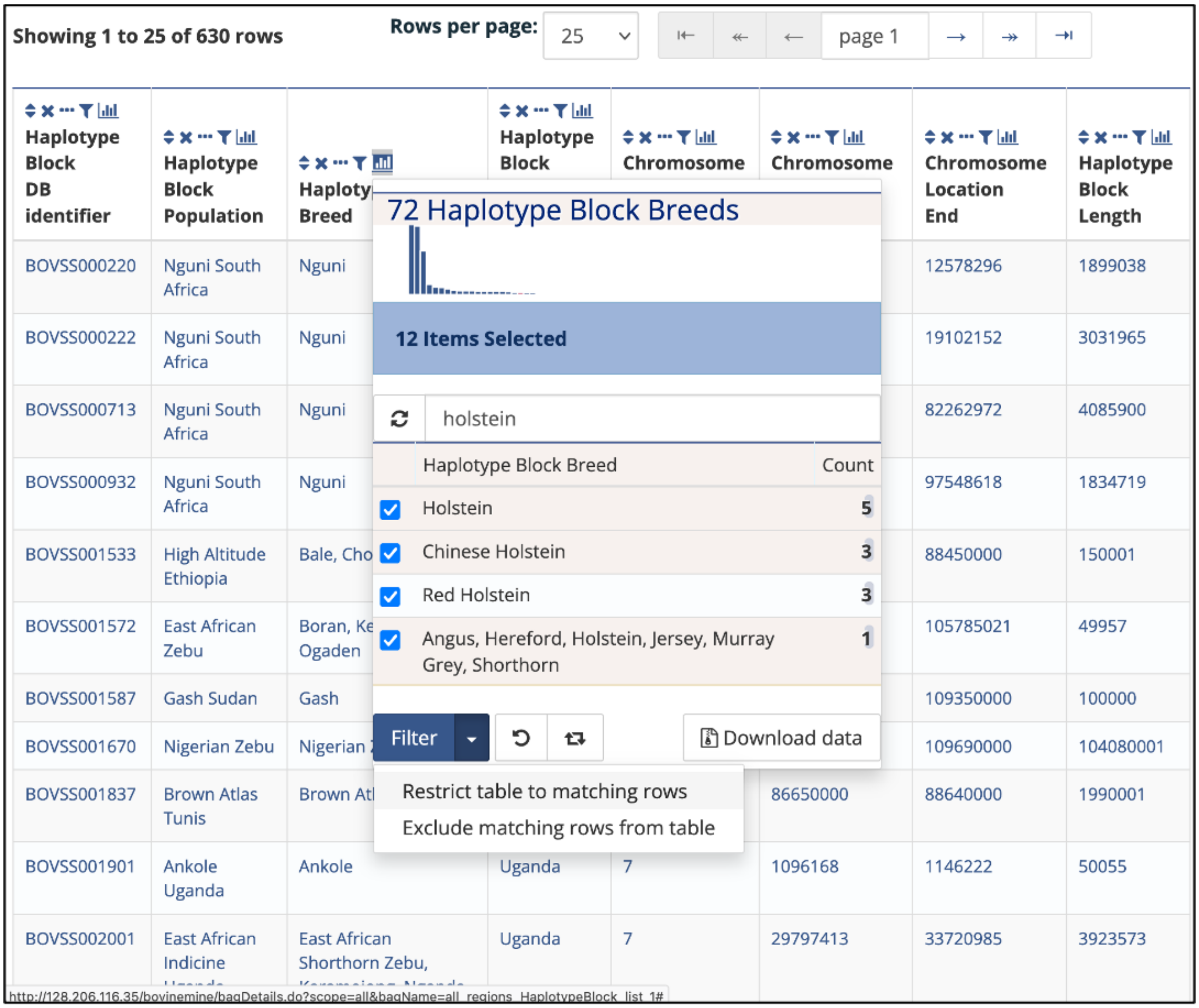

Figure S25. Filtering for selective sweeps in studies that include Holstein breeds.

Save the Holstein selective sweeps as a list for further analysis using steps like those described in Example 1.

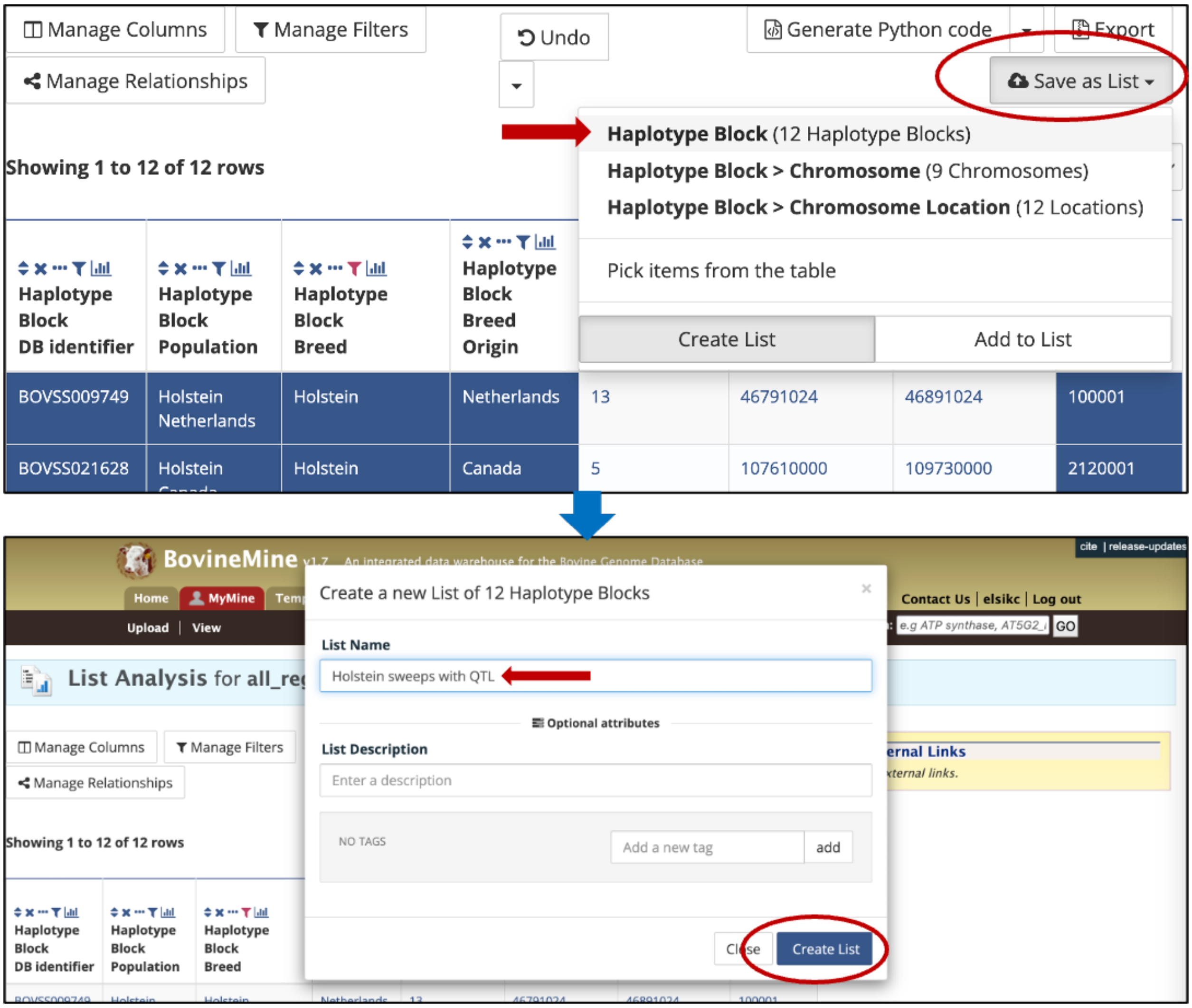

Figure S26. Saving Holstein selective sweeps that overlap QTL.

The saved list is used as input to the 'Selective Sweep (Haplotype Block) ID → Genes' query template to retrieve genes within the selective sweeps that overlap QTL.

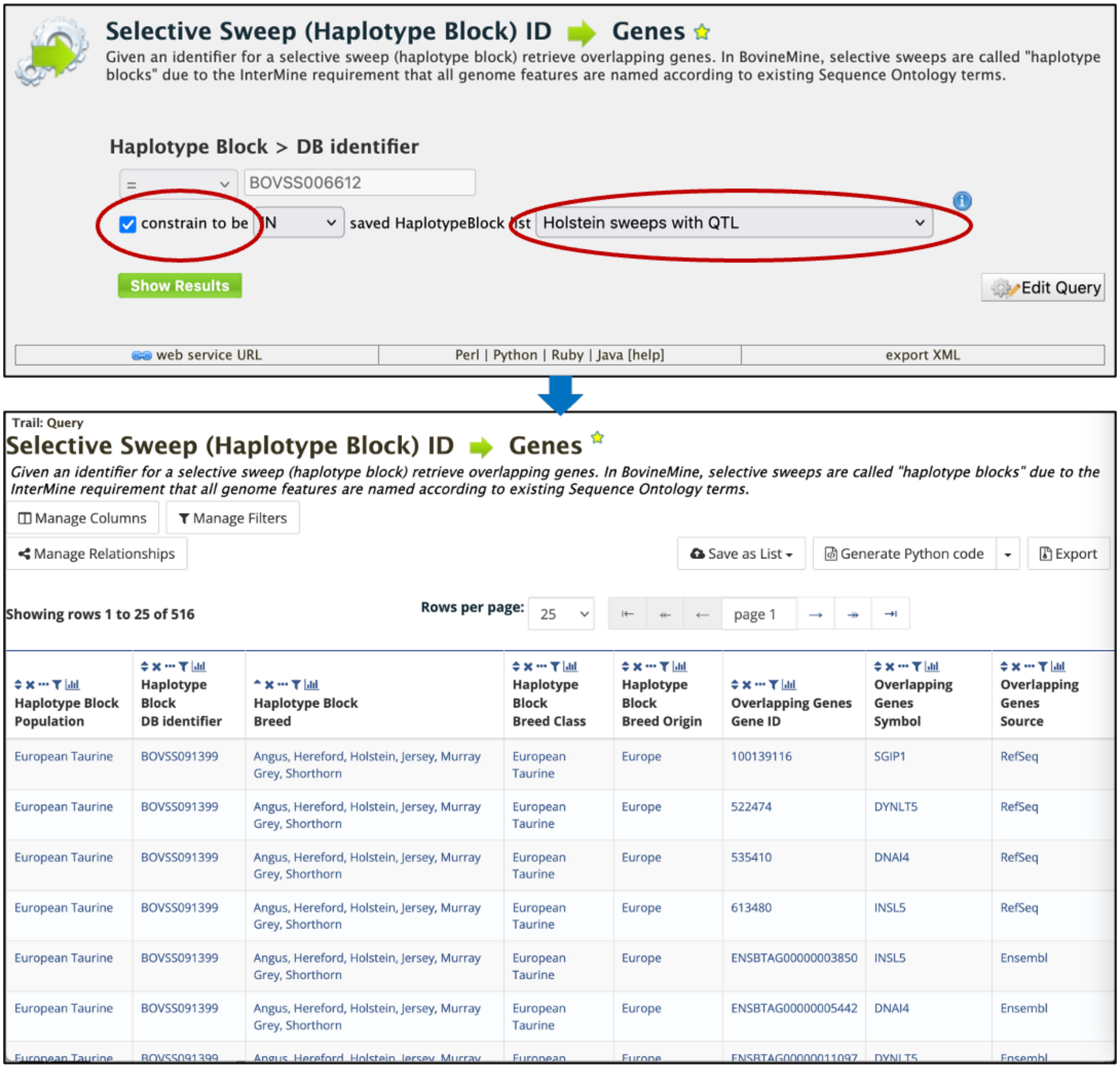

Figure S27. Using the template query 'Selective Sweep (Haplotype Block) ID → Genes' constrained with the list of Holstein selective sweeps harboring QTL.

References

1. Foll, M. and Gaggiotti, O. (2008) A genome-scan method to identify selected loci appropriate for both dominant and codominant markers: a Bayesian perspective. *Genetics*, **180**, 977–993.
2. Nielsen, R., Williamson, S., Kim, Y., Hubisz, M.J., Clark, A.G. and Bustamante, C. (2005) Genomic scans for selective sweeps using SNP data. *Genome Res*, **15**, 1566–1575.
3. Randhawa, I.A., Khatkar, M.S., Thomson, P.C. and Raadsma, H.W. (2014) Composite selection signals can localize the trait specific genomic regions in multi-breed populations of cattle and sheep. *BMC Genet*, **15**, 34.
4. Lotterhos, K.E., Card, D.C., Schaal, S.M., Wang, L., Collins, C. and Verity, B. (2017) Composite measures of selection can improve the signal-to-noise ratio in genome scans. *Methods in Ecology and Evolution*, **8**, 717–727.
5. Carneiro, M., Rubin, C.J., Di Palma, F., Albert, F.W., Alfoldi, J., Martinez Barrio, A., Pielberg, G., Rafati, N., Sayyab, S., Turner-Maier, J. *et al.* (2014) Rabbit genome analysis reveals a polygenic basis for phenotypic change during domestication. *Science*, **345**, 1074–1079.
6. Fu, Y.X. and Li, W.H. (1993) Statistical tests of neutrality of mutations. *Genetics*, **133**, 693–709.
7. Akey, J.M., Ruhe, A.L., Akey, D.T., Wong, A.K., Connelly, C.F., Madeoy, J., Nicholas, T.J. and Neff, M.W. (2010) Tracking footprints of artificial selection in the dog genome. *Proc Natl Acad Sci U S A*, **107**, 1160–1165.
8. Sabeti, P.C., Reich, D.E., Higgins, J.M., Levine, H.Z., Richter, D.J., Schaffner, S.F., Gabriel, S.B., Platko, J.V., Patterson, N.J., McDonald, G.J. *et al.* (2002) Detecting recent positive selection in the human genome from haplotype structure. *Nature*, **419**, 832–837.
9. Ramey, H.R., Decker, J.E., McKay, S.D., Rolf, M.M., Schnabel, R.D. and Taylor, J.F. (2013) Detection of selective sweeps in cattle using genome-wide SNP data. *BMC Genomics*, **14**, 382.
10. Weir, B.S. and Cockerham, C.C. (1984) Estimating F-Statistics for the Analysis of Population Structure. *Evolution*, **38**, 1358–1370.
11. Fariello, M.I., Servin, B., Tosser-Klopp, G., Rupp, R., Moreno, C., International Sheep Genomics, C., San Cristobal, M. and Boitard, S. (2014) Selection signatures in worldwide sheep populations. *PLoS One*, **9**, e103813.
12. Williams, J.L., Hall, S.J., Del Corvo, M., Ballingall, K.T., Colli, L., Ajmone Marsan, P. and Biscarini, F. (2016) Inbreeding and purging at the genomic Level: the Chillingham cattle reveal extensive, non-random SNP heterozygosity. *Anim Genet*, **47**, 19–27.
13. Boitard, S., Schlotterer, C. and Futschik, A. (2009) Detecting selective sweeps: a new approach based on hidden markov models. *Genetics*, **181**, 1567–1578.

14. Rubin, C.J., Zody, M.C., Eriksson, J., Meadows, J.R., Sherwood, E., Webster, M.T., Jiang, L., Ingman, M., Sharpe, T., Ka, S. *et al.* (2010) Whole-genome resequencing reveals loci under selection during chicken domestication. *Nature*, **464**, 587–591.
15. Voight, B.F., Kudaravalli, S., Wen, X. and Pritchard, J.K. (2006) A map of recent positive selection in the human genome. *PLoS Biol*, **4**, e72.
16. Vitalis, R., Gautier, M., Dawson, K.J. and Beaumont, M.A. (2014) Detecting and measuring selection from gene frequency data. *Genetics*, **196**, 799–817.
17. Kim, Y. and Nielsen, R. (2004) Linkage disequilibrium as a signature of selective sweeps. *Genetics*, **167**, 1513–1524.
18. Whitlock, M.C. (2005) Combining probability from independent tests: the weighted Z-method is superior to Fisher's approach. *J Evol Biol*, **18**, 1368–1373.
19. Ferrer-Admetlla, A., Liang, M., Korneliussen, T. and Nielsen, R. (2014) On detecting incomplete soft or hard selective sweeps using haplotype structure. *Mol Biol Evol*, **31**, 1275–1291.
20. Yi, X., Liang, Y., Huerta-Sanchez, E., Jin, X., Cuo, Z.X., Pool, J.E., Xu, X., Jiang, H., Vinckenbosch, N., Korneliussen, T.S. *et al.* (2010) Sequencing of 50 human exomes reveals adaptation to high altitude. *Science*, **329**, 75–78.
21. Luu, K., Bazin, E. and Blum, M.G. (2017) pcadapt: an R package to perform genome scans for selection based on principal component analysis. *Mol Ecol Resour*, **17**, 67–77.
22. Nei, M. and Li, W.H. (1979) Mathematical model for studying genetic variation in terms of restriction endonucleases. *Proc Natl Acad Sci U S A*, **76**, 5269–5273.
23. Ma, Y., Ding, X., Qanbari, S., Weigend, S., Zhang, Q. and Simianer, H. (2015) Properties of different selection signature statistics and a new strategy for combining them. *Heredity (Edinb)*, **115**, 426–436.
24. Tang, K., Thornton, K.R. and Stoneking, M. (2007) A new approach for using genome scans to detect recent positive selection in the human genome. *PLoS Biol*, **5**, e171.
25. Hosokawa, D., Ishii, A., Yamaji, K., Sasazaki, S., Oyama, K. and Mannen, H. (2012) Identification of divergently selected regions between Japanese Black and Holstein cattle using bovine 50k SNP array. *Anim Sci J*, **83**, 7–13.
26. Tajima, F. (1989) Statistical method for testing the neutral mutation hypothesis by DNA polymorphism. *Genetics*, **123**, 585–595.
27. Chen, H., Patterson, N. and Reich, D. (2010) Population differentiation as a test for selective sweeps. *Genome Res*, **20**, 393–402.
28. Sabeti, P.C., Varilly, P., Fry, B., Lohmueller, J., Hostetter, E., Cotsapas, C., Xie, X., Byrne, E.H., McCarroll, S.A., Gaudet, R. *et al.* (2007) Genome-wide detection and characterization of positive selection in human populations. *Nature*, **449**, 913–918.
29. Sousa Junior, L.P.B., Pinto, L.F.B., Cruz, V.A.R., Oliveira Junior, G.A., Oliveira, H.R., Chud, T.S., Pedrosa, V.B., Miglior, F., Schenkel, F.S. and Brito, L.F. (2024) Genome-wide association and functional genomic analyses for body conformation traits in North American Holstein cattle. *Front Genet*, **15**, 1478788.
